# Spontaneous vortex formation by microswimmers with retarded attractions

**DOI:** 10.1038/s41467-022-35427-7

**Published:** 2023-01-04

**Authors:** Xiangzun Wang, Pin-Chuan Chen, Klaus Kroy, Viktor Holubec, Frank Cichos

**Affiliations:** 1 Peter Debye Institute for Soft Matter Physics, Leipzig University, 04103 Leipzig, Germany; 2 Institute for Theoretical Physics, Leipzig University, Postfach 100 920, 04009 Leipzig, Germany; 3 Department of Macromolecular Physics, Faculty of Mathematics and Physics, Charles University, 18000 Prague, Czech Republic

**Keywords:** Phase transitions and critical phenomena, Statistical physics, Colloids

## Abstract

Collective states of inanimate particles self-assemble through physical interactions and thermal motion. Despite some phenomenological resemblance, including signatures of criticality, the autonomous dynamics that binds motile agents into flocks, herds, or swarms allows for much richer behavior. Low-dimensional models have hinted at the crucial role played in this respect by perceived information, decision-making, and feedback, implying that the corresponding interactions are inevitably retarded. Here we present experiments on spherical Brownian microswimmers with delayed self-propulsion toward a spatially fixed target. We observe a spontaneous symmetry breaking to a transiently chiral dynamical state and concomitant critical behavior that do not rely on many-particle cooperativity. By comparison with the stochastic delay differential equation of motion of a single swimmer, we pinpoint the delay-induced effective synchronization of the swimmers with their own past as the key mechanism. Increasing numbers of swimmers self-organize into layers with pro- and retrograde orbital motion, synchronized and stabilized by steric, phoretic, and hydrodynamic interactions. Our results demonstrate how even most simple retarded interactions can foster emergent complex adaptive behavior in small active-particle ensembles.

## Abstract (web summary)

Time-delayed interactions involving perception, decision, and reaction, are omnipresent in the living world. Here, the delayed self-propulsion of a microswimmer toward a target gives rise to chiral orbital motion via a symmetry-breaking bifurcation. Additional swimmers synchronize and stabilize it.

## Introduction

Ordered dynamical phases of motile organisms are ubiquitous in nature across all scales^1^, from bacterial colonies to insect swarms, and bird flocks^2^. In particular, self-organization into vortex patterns is often observed and has been attributed to some local external attractor, e.g., light or nutrient concentration, together with behavioral rules like collision avoidance and mutual alignment^3^. The pertinent social interactions are commonly thought to be based on perception^4–6^ and the ability to actively control the direction of motion^3^. They are also generally presumed to provide some benefits to the individual and to the collective, as in the case of collision avoidance or predator evasion^7,8^. However, since such interactions are usually derived only indirectly and approximately from observations^9^, it is arguably useful to coarse grain them, e.g., into simple alignment rules, in order to rationalize the collective effects with the help of simple mechanistic models, in particular with respect to their emerging universal traits^3,10–12^. This strategy has been successful in physics and is also supported by the observation that biological collectives often appear highly susceptible to environmental influences and exhibit a dynamical finite-size scaling reminiscent of critical states in inanimate many-body assemblies^13–16^.

Importantly, the cascades of complex biochemical/biophysical processes^17,18^ needed to transform signal perception into a navigational reaction inevitably result in retarded interactions upon coarse-graining^19^ (cf. supplementary Table S1). This generic complication is often dismissed in the analysis, and dedicated models and experiments addressing the role of time delays in the active matter are still rare^20–23^, although these have occasionally been shown to fundamentally alter the collective dynamics^21^ and to bring it closer to that found in nature^24^. To a first approximation, delay effects can resemble inertial corrections to an otherwise overdamped biological dynamics^25^. In particular, both have the propensity to give rise to oscillations and inertia, moreover, to rotational motion around an attractive center, as familiar from planetary orbits.

Experiments that can assess or even deliberately control retarded interactions in living systems turn out to be difficult. But by imposing time delays onto synthetic active particles via computer-controlled laser activation we can create an ideal laboratory system to experimentally emulate such situations. Suitable feedback control techniques for active particles have recently become available through photon nudging^26^. The technique allows to adjust a particle’s propulsion speed to acquire real-time information (positions and directions of motion) about the dynamical state of an ensemble. It has previously been employed to rectify the rotational Brownian motion for particle steering and trapping^27^, to explore orientation-density patterns in activity landscapes^28^, and to study information flow between active particles^23^, and their emerging critical states^29,30^. Beyond what related computer simulations accomplish^31–33^, these experiments additionally incorporate the full real-world complexity arising from actual physical interactions due to hydrodynamic, thermal, or concentration fields. In the following, we describe experiments with feedback-controlled active Brownian microswimmers aiming at a fixed target by a retarded thermophoretic self-propulsion. The systematic navigational errors resulting from the retardation are seen to cause a spontaneous symmetry breaking to a bi-stable dynamical state, in which the swimmers self-organize into a merry-go-round motion that switches transiently between degenerate chiralities.

## Results

### Single-particle retarded interaction

**Fig. 1:**
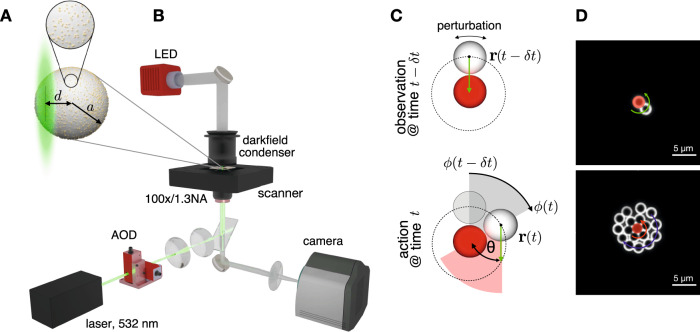
Experimental realization. **A** Particles used in the experiments consist of a melamine resin colloid (2.18 μm in diameter) with 8 nm gold nanoparticles scattered across the surface (covering up to 10% of the total surface area). A 532 nm laser focused at the edge of the particle at a distance *d* from its center induces a self-thermophoretic motion and allows for precise control of the propulsion direction. Importantly, optical forces are weak so the particles exhibit a truly self-phoretic autonomous motility, making them proper microswimmers. **B** Experimental setup used to image the particles by darkfield microscopy (LED, darkfield condenser, and camera) and guide their motion by sequential beam steering of the laser on the sample plane with a two-axis acousto-optic deflector (AOD). All particles in the field of view are addressed during each exposure period of the camera. **C** The interaction rule for the delayed attraction of a single active particle (white sphere) towards a target (red sphere) is split into an observation made at a time *t* − *δt* that sets the direction of motion for the self-propulsion step exerted after a programmed delay time *δt*. The green arrows indicate the planned motion −**r**(*t* − *δt*) and its actual realization at time *t*. **D** Examples of darkfield microscopy images where a single active particle (top) and 16 active particles (bottom) interact with one target particle (red).

The elementary component of a swarm is a single active particle whose direction of motion depends dynamically on its environment. Even small fluctuations of the particle position and orientation render any prospective active motion based on the perception of the environment inaccurate, due to the inevitable finite perception–action delay. In the most symmetric setup, an active particle moves toward a target position, which is occupied by an immobile particle of the same size in our experiments. Assuming that the active particle responds to the environment that was perceived a delay time *δt* earlier, its propulsion direction $\hat{{{{{{{{\bf{u}}}}}}}}}(t)$ at time *t* is determined by its relative position to the target particle at time *t* − *δt* in the past, according to

1 $$\hat{{{{{{{{\bf{u}}}}}}}}}(t)=\frac{-{{{{{{{\bf{r}}}}}}}}(t-\delta t)}{|{{{{{{{\bf{r}}}}}}}}(t-\delta t)|},$$

where **r** is the location of the active particle with respect to the target particle’s center. We implemented this interaction rule in an experimental feedback system that controls the active particles’ self-propulsion. Our active particles are polymer spheres of radius *a* = 1.09 μm, decorated with gold nanoparticles and suspended in a thin film of water. Laser light with a wavelength of 532 nm is focused at distance *d* from the active particle center (Fig. 1A). The resulting excentric heating excites an osmotic flow that lets the particle swim with a speed *v*_0_ in the direction defined by Eq. (1)^34^. A darkfield microscopy setup is used to image the particles (Fig. 1B). A computer analyzes and records the positions of the particles and then controls the laser position accordingly via an acousto-optic deflector. We use a separate calibrator particle running on a quadratic trajectory as a reference for the speed *v*_0_ attained by a free swimmer. Further details are described in Sec. 2 of the Supplementary Information.

**Fig. 2:**
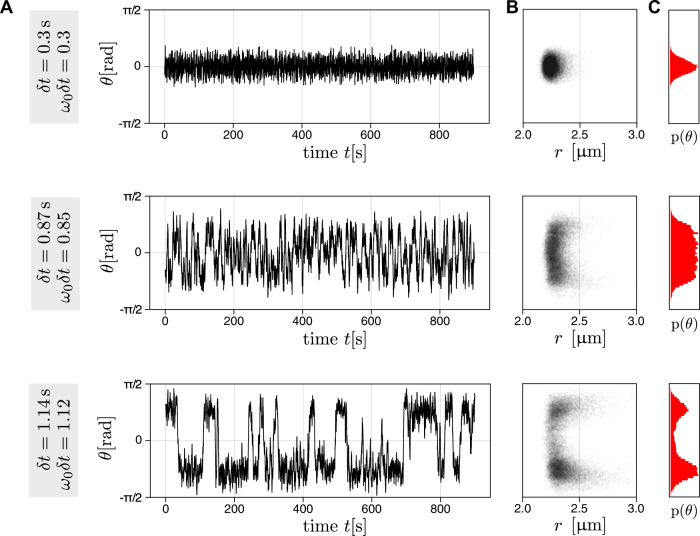
Propulsion angle at the different programmed delay. **A** Trajectories of the propulsion angle *θ*(*t*) of an active particle at three different delays (top: *δt* = 0.3 s, middle: *δt* = 0.87 s, and bottom: *δt* = 1.14 s) for its attraction towards a target particle. The velocity of the active particle is *v*_0_ = 2.16 μm s^−1^. **B** Propulsion angle *θ*(*t*) vs. the distance ∣**r**(*t*)∣ of the particle from the target center. **C** Histograms of the propulsion angle over the whole trajectory. The delay for the individual panels in columns (**B**, **C**) is indicated on the left of the corresponding row.

If *δt* = 0 s, the active particle moves towards the target particle until it collides with it. Further motion of the active particle is then constrained by the presence of the fixed target sphere, resulting in a diffusive motion around it, at a fluctuating distance consistent with the barometer formula^35,36^. As the delay *δt* increases, the diffusive motion induces a stochastic “error” component due to the increasingly misaligned self-propulsion. Once a critical delay is reached, the particle begins to orbit around the target (see Supplementary Movies 1–3). We quantify this dynamics by the angle *θ* between the direction of motion in Eq. (1) and the instantaneous negative radial direction −**r**(*t*) (see Fig. 2A). The angle *θ* itself or $\sin (\theta )$ can serve as an indicator for deviations from the “intended” central orientation. Similarly, for many particles, numbered by the index i, it is useful to define the rotational order parameters ${o}_{R,i}=({\hat{{{{{{{{\bf{r}}}}}}}}}}_{i}\times {\hat{{{{{{{{\bf{u}}}}}}}}}}_{i})\cdot {{{{{{{{\bf{e}}}}}}}}}_{z}=\sin ({\theta }_{i})$^29,37^, where the hats denote vectors normalized to 1 and **e**_z_ is a unit vector in the direction of z axis. Figure 2A shows the experimental trajectories of *θ* for a single active particle with *v*_0_ = 2.16 μm s^−1^ and three different delays. For short delays, *θ* fluctuates with a small amplitude around zero (Fig. 2A top). The fluctuations increase with the delay and lead to a flat-top probability density of the propulsion angle for *δt* ≈ 0.87 s (Fig. 2A middle). At larger delays (*δt* = 1.14 s), the propulsion angle fluctuates around a stable nonzero value that changes its sign intermittently (Fig. 2A bottom), corresponding to a bimodal probability density *p*(*θ*) (Fig. 2C). The periods of consistent chirality increase in duration when the delay is increased further. At *δt* = 1.4 s, the propulsion angle transiently fluctuates around ±80°. Under these conditions, the cohesion of the particle to the target becomes marginal as the typical particle velocity is almost tangential to the target particle circumference. As a result, the distance ∣**r**(*t*)∣ of the particle from the origin starts to fluctuate more strongly, as shown in the position histograms in Fig. 2B.

The net propulsion angle is the result of angular displacements *ϕ*(*t*) of the particle position acquired due to the perception–action delay during the period [*t* − *δt*, *t*]:

2 $$\theta (t)=\int\nolimits_{t-\delta t}^{t}\omega ({t}^{{\prime} })\,{{{{{{{\rm{d}}}}}}}}{t}^{{\prime} }=\phi (t)-\phi (t-\delta t)=\angle (\hat{{{{{{{{\bf{u}}}}}}}}}(t),- \, {{{{{{{\bf{r}}}}}}}}(t)).$$

**Fig. 3:**
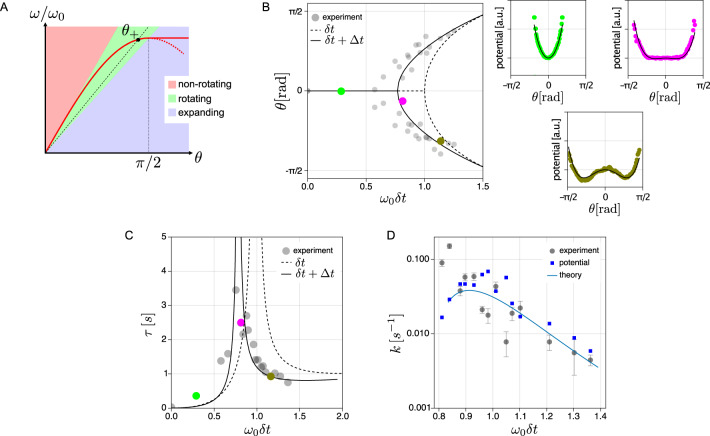
Transition to a rotational dynamical state for a single active particle. **A** Graphical construction of condition (3) for a transition from an non-rotational state (red-shaded region) to a rotational state (green-shaded region). The red line ($\sin \theta$) and the black dashed line with slope 1/(*ω*_0_*δt*) intersect at several *θ*. The solution *θ* = *θ*_+_ in the green region and its chirally inverse image *θ*_−_ in the third quadrant (not shown) correspond to co- and counter-clockwise rotation. **B** Experimentally measured propulsion angles (maxima of the histograms in Fig. 2C) as a function of *ω*_0_*δt*, exhibiting a bifurcation at *ω*_0_*δt* ≈ 0.76. The dashed line corresponds to the analytical prediction of the theoretical model (5), neglecting the inevitable instrumental delay Δ*t*. The solid line shows the solution of the refined theoretical model, which includes the instrumental delay Δ*t* = 64 ms of our setup in addition to the programmed delay *δt*. The colored dots indicate the control parameter values studied in Fig. 2 and the linked small color plots show the corresponding potentials of mean force, determined from the propulsion angle histograms in Fig. 2C, together with a fit of the refined analytical model, including the instrumental delay Δ*t* (see Sec. 2.2 and 3 of Supplementary Information). The only free parameter for fitting is the effective temperature of the system. **C** Relaxation time *τ* of a single active particle as determined experimentally from the autocorrelation of the propulsion angle fluctuations (Eq. (8), data points). The solid lines correspond to the refined version of the theoretical prediction (Eq. (7)), including the instrumental delay Δ*t* (see Sec. 2.2 of Supplementary Information for details). The colored dots have the same meaning as in panel (**B**). **D** Transition rates between the two rotational states obtained from the experiments (circles) plotted with the predictions from Kramers’ theory, Eq. (9), with a global fit parameter *D*_*θ*_ = 0.05 s^−1^ (solid line) and *D*_*θ*_ fitted to the probability distribution *p*(*θ*) separately for each value *ω*_0_*δt* (squares). Error bars represent the standard error.

Here, *ϕ*(*t*) is the polar angle of the active particle in polar coordinates centered in the target particle, and we introduced $\omega (t)=\dot{\phi }(t)$ as its corresponding angular velocity (Fig. 2C). The observed dynamics can be understood by considering the active particle and the target particle in physical contact. Their distance is then constrained to be the sum of their radii (*R* = 2*a* = 〈∣**r**(*t*)∣〉) and the active particle slides around the target particle with an angular velocity $\omega (t)={\omega }_{0}\sin (\theta (t))$, where *ω*_0_ = *v*_0_/*R* is the natural angular velocity for tangential propulsion with *θ* = ±*π*/2. As sketched in Fig. 3A, assuming a constant angular velocity *ω* with *θ* = *ωδt*, the solutions to the equation for *θ* are given by the intersections of a sine function and a linear function,

3 $${({\omega }_{0}\delta t)}^{-1}\theta \,=\sin (\theta ).$$

For *ω*_0_*δt* < 1, there is a single intersection at *θ* = 0, indicating a stable non-rotational state. For 1 < *ω*_0_*δt* < *π*/2, the non-rotational state becomes unstable and two counter-rotational metastable solutions arise. For *ω*_0_*δt* > *π*/2, the rotating solutions correspond to ∣*θ*∣ > *π*/2, and the radial component of propulsion becomes positive (repulsive), driving the active particle away from the target particle. As a result, the orbit “takes off” and its radius *R* increases until a new stable orbit with *R* = 2*v*_0_*δt*/*π* > 2*a* and ∣*θ*∣ = *π*/2 is reached. For small particles (*a* → 0), the distance of the swimmer to the target position can thus, in principle, vanish (*R* → 0), and the rotating orbits can even occur at arbitrarily short programmed delays (*δt* → 0). Retarded attraction hence always leads to rotational orbital motion with a delay-dependent radius^23^. In the experiment, due to the presence of the fixed central particle, the smallest attainable orbit radius *R* = 2*a* is given by the particle diameter. Adding Brownian fluctuations to the deterministic Eq. (3) results in the nonlinear delayed stochastic differential equation $\dot{\phi }(t)={\omega }_{0} \sin \left(\phi (t)- \phi (t-\delta t)\right) +\scriptstyle\sqrt{2{D}_{0}/{R}^{2}}\,\eta (t)$, where *D*_0_ ≈ 0.0642 μm^2^ s^−1^ denotes the translational diffusion coefficient of the active particle and *η*(*t*) white noise. To solve this equation, we approximated $\dot{\phi }(t)\delta t$ by *θ*(t) and expanded the $\sin (\phi (t)-\phi (t-\delta t))$ in a Taylor series around *δt* = 0 up to the third order in *δt*. We dropped the term proportional to *ϕ⃛*(*t*) to secure the stability of the resulting equation^38^ (for details, see Sec. 3 of Supplementary Information). The resulting noise term $\scriptstyle\sqrt{8{D}_{0}/{({\omega }_{0}\delta tR)}^{2}}$ turned out to be inaccurate compared to experimental and simulation data. We, therefore, introduce an effective diffusion coefficient *D*_*θ*_ as a free parameter in the noise term in Eq. (4) to describe the rotation of the active particle around the target as the angular Brownian motion

4 $$\dot{\theta }=\frac{1}{3\delta t}\left[{\theta }_{\pm }^{2}-{\theta }^{2}\right]\theta+\sqrt{2{D}_{\theta }}\eta$$

with

5 $${\theta }_{\pm }=\pm \sqrt{6\left(1-\frac{1}{{\omega }_{0}\delta t}\right)}.$$

Eq. (4) yields the stationary solutions 0 and *θ*_±_ with the bifurcation point *ω*_0_*δt* = 1, for the transition from a non-rotational to a rotational state. The data points in Fig. 3B display the experimentally obtained maxima of the histograms *p*(*θ*) of the propulsion angle (see Fig. 2C) as a function of *ω*_0_*δt*. The transition points in the experiments are located at lower values of the control parameter *ω*_0_*δt*, due to the mentioned instrumental delay Δ*t* in the feedback loop of the experimental setup. This instrumental delay between the most recent exposure to the camera and the laser positioning affects the motion direction beyond the programmed delay *δt*^34,39^, causing an earlier onset of the transition to a stable rotation. The dashed line in Fig. 3B shows the theoretical prediction, which includes both the instrumental delay Δ*t* and the programmed delay *δt*, as detailed in the Supplementary Information (Eq. (11)).

The Langevin equation (4) can be interpreted as a dynamical equation for the position *θ* of an overdamped Brownian particle with diffusion coefficient *D*_*θ*_ in a quartic potential (see derivation in Sec. 3 of Supplementary Information),

6 $$U(\theta )=\frac{1}{\delta t}\left[\left(\frac{1}{{\omega }_{0}\delta t}-1\right){\theta }^{2}+\frac{1}{12}{\theta }^{4}\right],$$

which allows to classify the observed instability of the isotropic state as a normal supercritical pitchfork bifurcation^40^. The potential can also directly be extracted from the experimental data (Fig. 3B) by fitting the histogram *p*(*θ*) with a (normalized) Boltzmann distribution $\exp (-U(\theta )/{D}_{\theta })/Z$ at the effective temperature *D*_*θ*_. The effective temperature thus links the measured potential of mean force $-{D}_{\theta }\log p(\theta )$ to Eq. (6).

The latter resembles the Landau free energy at a second-order phase transition^41^. For readers familiar with this framework, this mathematical analogy allows to shortcut the following analysis, the details of which are given in Sec. 3 of the Supplementary Information. Note, however, that we are not discussing a thermodynamic phase transition but merely a dynamical bifurcation, here. The bifurcation and its potential energy landscape are not due to strong many-particle couplings, but to the interaction of the single active particle with its own past image. In Landau’s theory, the control parameter 1 − *ω*_0_*δt* maps onto the dimensionless distance to the critical temperature. Both the activity *ω*_0_ and the delay *δt* favor the transition to the symmetry-broken state. Hence, at high propulsion speeds, already short delays can give rise to rotating orbits. The inverse of the second derivative of *U*(*θ*), corresponding to the static susceptibility in Landau theory, gives the time *τ* (Eq. (7)) to relax in the (meta-)stable states,

7 $$\tau=\left\{\begin{array}{ll}\frac{\delta t}{2}{\left(\frac{1}{{\omega }_{0}\delta t}-1\right)}^{-1} &{\omega }_{0}\delta t \, < \,1\\ -\frac{\delta t}{4}{\left(\frac{1}{{\omega }_{0}\delta t}-1\right)}^{-1} &{\omega }_{0}\delta t \, > \,1.\end{array}\right.$$

We determine *τ* experimentally via *C*(*τ*) from the autocorrelation function,

8 $$C(t)=\frac{{\langle \delta \theta ({t}^{{\prime} }+t)\delta \theta (t)\rangle }_{{t}^{{\prime} }}}{{\langle \delta \theta {({t}^{{\prime} })}^{2}\rangle }_{{t}^{{\prime} }}}$$

of fluctuations of the propulsion angle *δθ*(*t*) = *θ*(*t*) − 〈*θ*(*t*)〉, as *C*(*τ*) = 1/e (Fig. 3C). The experimental data (circles) is compared to Eq. (7) (dashed line), and to an improved model prediction (solid line) that also takes into account the inevitable instrumental delay Δ*t*, as discussed in Sec. 3 of the Supplementary Information. The critical slowing down of the relaxation due to an increasingly flat potential close to the transition point at *ω*_0_*δt* = 1, corresponding to the potential plot in the middle of Fig. 3B, is thereby nicely confirmed, without any free parameter.

While the rotational orbits can be inferred from a purely deterministic model excluding Brownian motion, the observed spontaneous reversal of the chirality is driven by fluctuations in the propulsion angle and, thus, by the (non-equilibrium) noise in the system. It corresponds to transitions between the minima ±*θ*_±_ of the virtual potential, Eq. (6). We may thus apply Kramers’ theory to estimate the corresponding transition rate as

9 $$k=\frac{\sqrt{2}}{\pi }\frac{|{\omega }_{0}\delta t-1|}{{\omega }_{0}\delta {t}^{2}}\exp \left[-\frac{3}{\delta t{D}_{\theta }}{\left(\frac{1}{{\omega }_{0}\delta t}-1\right)}^{2}\right].$$

The effective temperature *D*_*θ*_ driving the fluctuations in the virtual potential is treated as a fit parameter. Figure 3D displays the experimentally measured transition rates, obtained from the observed mean residence times of *θ* in the two potential wells. They are in good agreement with Eq. (9), despite the hybrid equilibrium/non-equilibrium origin of the noisy dynamics.

### Multiple particles

As demonstrated in the previous section, the rotation observed in our experiments results from a spontaneous symmetry breaking in the dynamics of a single active particle. It originates from the particle’s retarded self-propulsion to a target, which differs from standard explanations of rotational dynamics in overdamped systems, which usually blame mutual (“social”) interactions between multiple agents^3,9,12,42^. As we demonstrate in Fig. S9B, when adding up to five more active particles to the system, each of them exhibits the same rotation and bifurcation as a single swimmer. Steric, hydrodynamic, and thermophoretic interactions among the particles then synchronize and stabilize their motion, aligning their sense of rotation. So the system exhibits collective behavior, but the dynamical symmetry breaking to a chiral dynamical state is not primarily due to the mutual interactions.

**Fig. 4:**
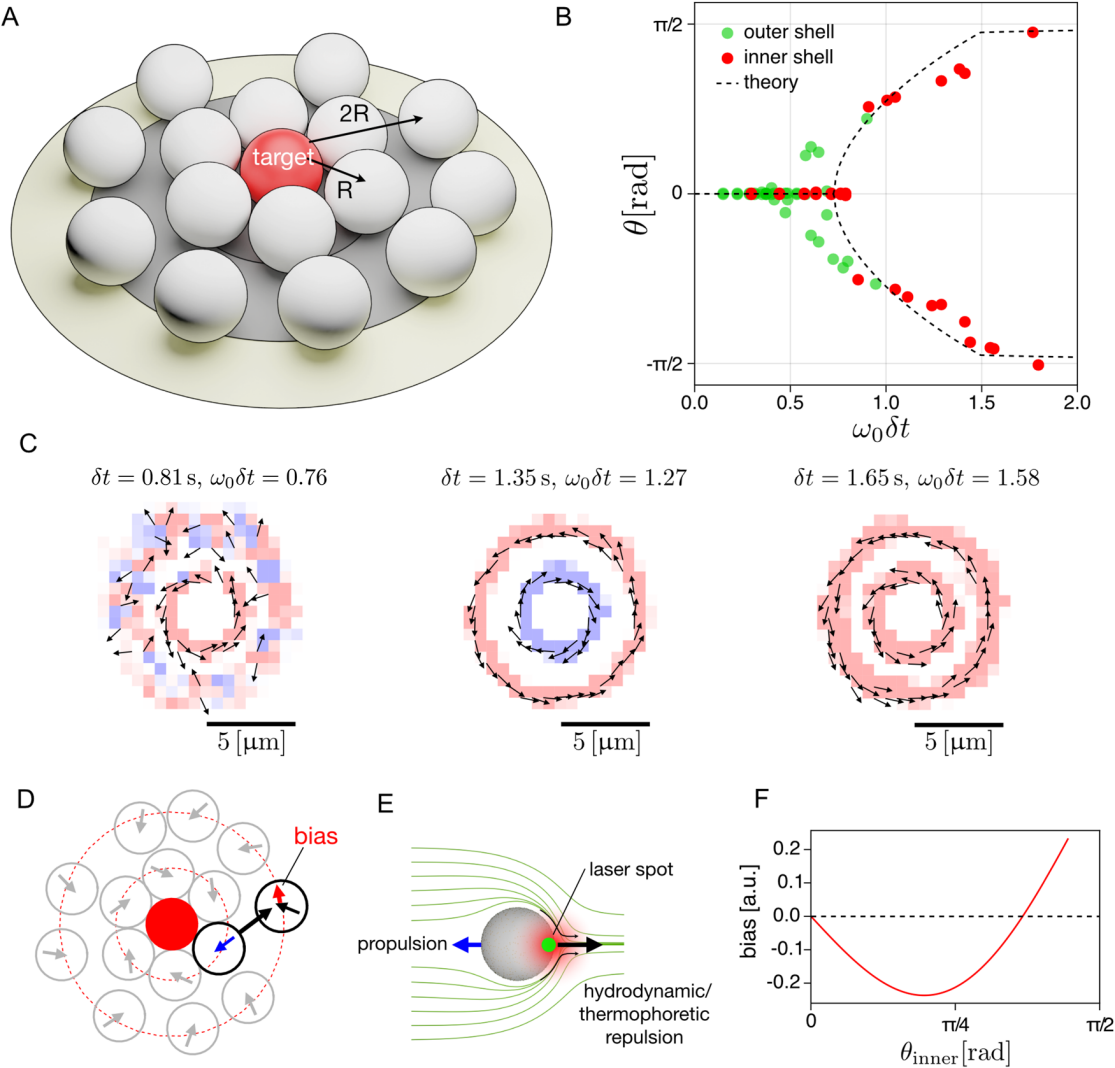
Collective rotation of 15 particles attracted to a single target particle. **A** Sketch of the shell structure and radii. **B** Bifurcation of the most probable propulsion angle as a function of the control parameter *ω*_0_*δt* for a (calibrator) propulsion speed of *v*_0_ = 2.06 μm s^−1^. The red dots are obtained from the inner shell particles at a typical distance of *R*^in^ = 2.18 μm, while the green dots denote the outer shell particles at *R*^out^ = 4.47 μm. The dashed line corresponds to the theoretical single-particle prediction, including the instrumental delay Δ*t* = 70 ms. **C** Average velocity field of active particles at *δt* = 0.81 s when the spontaneous rotation of the inner shell is constantly disrupted by the non-rotating outer shell, at *δt* = 1.35 s when the two shells are counter-rotating, and at *δt* = 1.65 s when both shells are co-rotating. The arrows and colors denote the average direction of motion. **D** Snapshot of the active particles and their propulsion directions corresponding to **(C)** at *δt* = 1.35 s. The repulsion induced by the flow and temperature fields of the inner shell causes a bias for the outer shell rotation. **E** Sketch of the flow and temperature fields induced by the laser (green dot) around an active particle, and the resulting repulsion. **F** Schematic sketch of the presumed magnitude of the bias caused by the temperature and flow fields on the rotation of the outer shell, as a function of the propulsion angle *θ*^in^ of the inner shell particles (see Sec. 5 of Supplementary Information).

Somewhat larger numbers of particles organize into multiple rotating shells. Figure 4 summarizes the key results obtained for an ensemble of 15 active particles attracted to the target particle with the same programmed and intrinsic delays *δt* and Δ*t*, respectively. For the considered range of time delays, the active particles form two tightly packed shells around the target particle (Fig. 4A). The typical distance of the inner shell particles to the target is about half that of the outer shell, *R*^out^ ≈ 2*R*^in^ = 4*a*. So based on the single-particle picture alone, the particles in the inner and outer shells swimming at the same speed would be expected to start rotating at different delays. However, in reality, the inter-particle interactions in the compact cluster strongly correlate with the particle motion and quantitatively change the picture. Compared to the theoretical prediction, *ω*_0_*δt* = 0.73, we observe that for *v*_0_ = 2.06 μm s^−1^ the transition to the rotational phase of the inner shell is postponed to ${\omega }_{0}^{{{{{{{{\rm{in}}}}}}}}}\delta t\equiv {v}_{0}\delta t/{R}^{{{{{{{{\rm{in}}}}}}}}}\,\approx \,$ 0.83, corresponding to *δt* = 0.9 s (see the rightmost red data point lying on the horizontal axis in Fig. 4B). Slightly below the transition, the inner shell exhibits alternating periods of rotational and stationary states. Meanwhile, the stationary outer shell compresses the inner shell due to its inwards-pointing propulsion direction (Fig. 4C, left). Figure. 4C displays the velocity fields of the particles averaged over their trajectories with three different delays. The bifurcation for the outer shell is located at ${\omega }_{0}^{{{{{{{{\rm{out}}}}}}}}}\delta t\equiv {v}_{0}\delta t/{R}^{{{{{{{{\rm{out}}}}}}}}}\,\approx\,$0.41, which corresponds to the same value *δt* = 0.9 s of the delay at which the inner shell undergoes its bifurcation to the rotational state (see Fig. 4B and Supplementary Movies S4–S6). For delays slightly above the transition, 0.9 s < *δt* < 1.41 s, the two shells rotate in opposite directions, as shown in the middle plot of Fig. 4C. The simultaneous transition and the counter-rotation of the two shells suggest that the inner shell particles generate backflows opposite to their propulsion direction, thereby repelling the outer shell particles and facilitating their transition to the rotational state, as schematically depicted in Fig. 4D–F. These backflows are presumably caused by the directional hydrodynamic and thermophoretic interactions. The surface temperature gradient across each particle creates a thermo-osmotic surface flow that propels the particle^43^. If the particle motion is opposed by an external force, such as the steric force due to the immobilized target particle, the slowed-down particle acts as a pump, creating a hydrodynamic outflow at its hot side (Fig. 4D and Sec. 2.5 and 5.2 of Supplementary Information). Furthermore, thermophoretic interactions arise from temperature gradients across the surface of a particle caused by its neighbors^33^. These are commonly repulsive, as found, e.g., for Janus particles in external temperature gradients^33^. We have carried out finite element simulations of the flow field around a mobile and an immobile self-propelling swimmer (see Sec. 2.5 of Supplementary Information). The overall near-field hydrodynamic interactions are found to be quite complex, due to many interacting particles and the nearby substrate surface^44–46^. They also depend on the propulsion angle *θ*. An increasing innershell propulsion angle results in a changing direction and magnitude of the rotational bias onto the outer shell, which presumably varies as sketched in Fig. 4F (see Sec. 5 of Supplementary Information). As a result, for *δt* ≥ 1.41 s, the two shells predominantly rotate in the same sense, as shown in Fig. 4C, right. The transition from counter- to co-rotation shells corresponds to the sign flip of the bias at *θ*^in^ ≈ 67^∘^. At even longer delays, *θ*^in^ tends to reach 90^∘^, and thus the inner shell tries to take off and expand against the compression exerted by the outer shell. These competing tendencies lead to particle exchange between the two shells. While we currently cannot separate thermophoretic and hydrodynamic effects in the experiment, hydrodynamic interactions may be expected to be more important here than for a single free particle in a temperature gradient: firstly, due to the collective character of the dynamics, and secondly, due to the pump effect caused by the partial blocking of the self-phoretic motion of the individual swimmers (see Sec. 2.5 and 5.2 of Supplementary Information). These features could provide a link between our experiments and the swarming observed in bacterial colonies^47,48^.

## Discussion

We have demonstrated above that the motion of an active particle induced by the delayed attraction to a target point can spontaneously undergo a transition from a diffuse isotropic “barometric” state to a dynamical chiral state, upon increasing the activity and/or the delay time. The transition is well described by a pitchfork bifurcation accompanied by a characteristic critical slowing down of the response^40^. Similar to certain mechanical analogs^49^, the single-particle dynamics thus already exhibit non-trivial features more commonly associated with (mean-field) phase transitions in strongly interacting passive many-body systems. This can be explained by noting that the deterministic part, $\dot{\phi }(t)={\omega }_{0}\sin \left(\phi (t)-\phi (t-\delta t)\right)$, of our stochastic delay differential equation can also be understood as the dynamical equation for a single Kuramoto phase oscillator^50,51^, with vanishing eigenfrequency and coupling strength *ω*_0_, which is trying to synchronize with its own past state. In the chiral state, the particle orbits around the target point (the central obstacle is optional). The orbiting motion is stable against noise, but its sense of rotation is only transiently maintained. This should be contrasted with the chiral states resulting from non-reciprocal coupling in the time-local Kuramoto model (without delay), as discussed by ref. 52, which hinges on the stabilization by many-body cooperativity. Based on our results, we suggest that for the single retarded oscillator, the infinite number of relaxation modes encoded in the time-delayed equation of motion play a similar role^53,54^.

As we have shown, the nonlinear dynamics of our experimental system can be described by an approximate analytical model, which explains the emergence of a self-generated quartic virtual potential. While such potentials are frequently found in descriptions of phase transitions and collective effects in active-particle ensembles, following various behavioral rules^29,30^, we reiterate that the mechanism is a different one, here. Due to the activity and the (programmed) delay, it already occurs for a single active particle aiming at a spatially fixed target. In a whole swarm of particles that are all attracted to a common target, which might be its own perceived center of mass, the single-particle bifurcation is preserved. Inter-particle collisions merely synchronize, renormalize, and stabilize the rotational states of the individual particles. Upon close contact, hydrodynamic and thermophoretic interactions become important and help the swimmers to self-organize into co- and counter-rotating orbits. In biological motile ensembles, from bacteria to fish, similar hydrodynamic mechanisms may be at work, although precise details and scales may differ widely^45,55–57^. The corresponding many-body effects can be subtle and may elude coarse-grained simulations and theories. This underscores the importance of well-controlled experimental model systems that may act as “hybrid simulations”, combining computer-controlled active particles with real-world environments.

To conclude, while time delays are an unavoidable outcome of coarse-graining microscopic descriptions of the feedback processes in natural systems (cf. Table S1), they are often neglected in low-dimensional models of active particle collective effects^5,10^. In this respect, our model system provides a new perspective, as it takes the unavoidable systematic delays in the dynamics seriously and explores their generic effects. We find that, in overdamped systems, retardation plays a similar role as added inertia. Both effects lead to persistence and associated “aiming errors” in particle dynamics. In this sense, our analysis can provide a template for an entire class of motile ensembles exhibiting spontaneous rotational dynamics caused by aiming errors—as such, are associated with microswimmer navigation strategies employing “vision-cone”^29,30^ or “acceptance-angle”^27,36^ criteria. In fact, the effects of the time delay may be even richer^20,24,54^. While we considered only a positive delay, i.e., synchronization with the past, above, sophisticated biological organisms also possess predictive capabilities to extrapolate the current state into the future^58,59^. These can, to a first approximation, be incorporated in the form of a negative time delay. The inclusion of positive and negative delays may therefore provide a new, “more physical” perspective on phenomenologically extracted, rather sophisticated rules like collision avoidance and alignment interactions, commonly postulated as sources of emerging complex adaptive responses in living many-body systems.

## Methods

### Sample preparation

Samples were prepared using two glass coverslips (20 mm × 20 mm, 24 mm × 24 mm) to confine a thin liquid layer (3 μm thickness) in between. The edges of one coverslip are sealed with a thin layer of PDMS (polydimethylsiloxane) to prevent leakage and evaporation. The liquid film used in the sample is composed of 2.19-μm-diameter gold-coated melamine formaldehyde (MF) particles (microParticles GmbH) dispersed in 0.1% Pluronic F-127 solution. The latter prevents the cohesion of the particles and adsorption to the cover slide surface. The surface of the MF particles is speckled uniformly with gold nanoparticles of about 8 nm diameter with a total surface coverage of about 10% (Fig. S3A). SiO_2_ particles (2.96 μm in diameter, microParticles GmbH) are added into the solution to keep the thickness of the liquid layer at about 3 μm. Finally, 0.3 μl of the mixed particle suspension is pipetted on one of the coverslips, for which the other serves as a lid.

### Experimental setup

The experimental setup (see Sec. 2 of Supplementary Information) consists of an inverted microscope (Olympus, IX71) with a mounted piezo translation stage (Physik Instrumente, P-733.3). The sample is illuminated with an oil-immersion darkfield condenser (Olympus, U-DCW, NA 1.2–1.4) and a white-light LED (Thorlabs, SOLIS-3C). The scattered light is imaged by an objective lens (Olympus, UPlanApo × 100/1.35, Oil, Iris, NA 0.5–1.35) and a tube lens (250 mm) to an EMCCD (electron-multiplying charge-coupled device) camera (Andor, iXon DV885LC). The variable numerical aperture of the objective was set to a value below the minimum aperture of the darkfield condenser.

The microparticles are heated by a focused, continuous-wave laser at a wavelength of 532 nm (CNI, MGL-III-532). The beam diameter is increased by a beam expander and sent to an acousto-optic deflector (AA Opto-Electronic, DTSXY-400-532) and a lens system to steer the laser focus in the sample plane. The deflected beam is directed towards the sample by a dichroic beam splitter (D, Omega Optical, 560DRLP) and focused by an oil-immersion objective (Olympus, UPlanApo × 100/1.35, Oil, Iris, NA 0.5–1.35) to the sample plane (*w*_0_ ≈ 0.8 μm beam waist in the sample plane). A notch filter (Thorlabs, NF533-17) is used to block any remaining back reflections of the laser from the detection path. The acousto-optic deflector (AOD), as well as the piezo stage, are driven by an AD/DA (analog-digital/digital-analog) converter (Jäger Messtechnik, ADwin-Gold II). A LabVIEW program running on a desktop PC (Intel Core i7 2600 4 × 3.40 GHz CPU) is used to record and process the images as well as to control the AOD feedback via the AD/DA converter.

## Supplementary information

Supplementary Information

Peer Review File

Description of Additional Supplementary Files

Supplementary Video 1

Supplementary Video 2

Supplementary Video 3

Supplementary Video 4

Supplementary Video 5

Supplementary Video 6

## Data Availability

All data in support of this work is available in the manuscript or the Supplementary Information. Further data and materials are available from the corresponding author upon request.

## References

[1] Kauffman, S. *The Origins of Order: Self-organization and Selection in Evolution* (Oxford Univ. Press, 1993).

[2] Vicsek T, Zafeiris A (2012). Collective motion. Phys. Rep..

[3] Delcourt J, Bode NWF, Denoël M (2016). Collective vortex behaviors: diversity, proximate, and ultimate causes of circular animal group movements. Q. Rev. Biol..

[4] Strandburg-Peshkin A (2013). Visual sensory networks and effective information transfer in animal groups. Curr. Biol..

[5] Pearce DJG, Miller AM, Rowlands G, Turner MS (2014). Role of projection in the control of bird flocks. Proc. Natl. Acad. Sci. USA.

[6] Cremer J (2019). Chemotaxis as a navigation strategy to boost range expansion. Nature.

[7] Couzin, I. D. & Krause, J. Self-organization and collective behavior in vertebrates. *Adv. Study Behav*. **32**, 1–75 (2003).

[8] Ioannou CC, Guttal V, Couzin ID (2012). Predatory fish select for coordinated collective motion in virtual Prey. Science.

[9] Berdahl AM (2018). Collective animal navigation and migratory culture: from theoretical models to empirical evidence. Philos. Trans. R. Soc. B Biol. Sci..

[10] Vicsek T, Czirók A, Ben-Jacob E, Cohen I, Shochet O (1995). Novel type of phase transition in a system of self-driven particles. Phys. Rev. Lett..

[11] Hemelrijk CK, Hildenbrandt H (2011). Some causes of the variable shape of flocks of birds. PLoS ONE.

[12] Costanzo A, Hemelrijk CK (2018). Spontaneous emergence of milling (vortex state) in a Vicsek-like model. J. Phys. D Appl. Phys..

[13] Cavagna A (2010). Scale-free correlations in starling flocks. Proc. Natl. Acad. Sci. USA.

[14] Mora T, Bialek W (2011). Are biological systems poised at criticality?. J. Stat. Phys..

[15] Muñoz MA (2018). Colloquium: criticality and dynamical scaling in living systems. Rev. Mod. Phys..

[16] Cavagna A (2017). Dynamic scaling in natural swarms. Nat. Phys..

[17] Kim, D. W., Hong, H. & Kim, J. K. Systematic inference identifies a major source of heterogeneity in cell signaling dynamics: the rate-limiting step number. *Sci. Adv*. **8**, eabl4598 (2022). 10.1126/sciadv.abl4598 PMC8932658 35302852

[18] Zhang J, Zhou T (2019). Markovian approaches to modeling intracellular reaction processes with molecular memory. Proc. Natl. Acad. Sci. USA.

[19] More HL, Donelan JM (2018). Scaling of sensorimotor delays in terrestrial mammals. Proc. R. Soc. B Biol..

[20] Mijalkov M, McDaniel A, Wehr J, Volpe G (2016). Engineering sensorial delay to control phototaxis and emergent collective behaviors. Phys. Rev. X.

[21] Forgoston E, Schwartz IB (2008). Delay-induced instabilities in self-propelling swarms. Phys. Rev. E.

[22] Piwowarczyk R, Selin M, Ihle T, Volpe G (2019). Influence of sensorial delay on clustering and swarming. Phys. Rev. E.

[23] Khadka U, Holubec V, Yang H, Cichos F (2018). Active particles bound by information flows. Nat. Commun..

[24] Holubec V, Geiss D, Loos SAM, Kroy K, Cichos F (2021). Finite-size scaling at the edge of disorder in a time-delay vicsek model. Phys. Rev. Lett..

[25] Attanasi A (2014). Information transfer and behavioural inertia in starling flocks. Nat. Phys..

[26] Qian B, Montiel D, Bregulla A, Cichos F, Yang H (2013). Harnessing thermal fluctuations for purposeful activities: The manipulation of single micro-swimmers by adaptive photon nudging. Chem. Sci..

[27] Bregulla AP, Yang H, Cichos F (2014). Stochastic localization of microswimmers by photon nudging. ACS Nano.

[28] Söker NA, Auschra S, Holubec V, Kroy K, Cichos F (2021). How activity landscapes polarize microswimmers without alignment forces. Phys. Rev. Lett..

[29] Baeuerle T, Loeffler RC, Bechinger C (2020). Formation of stable and responsive collective states in suspensions of active colloids. Nat. Commun..

[30] Loeffler RC, Baeuerle T, Kardar M, Rohwer CM, Bechinger C (2021). Behavior-dependent critical dynamics in collective states of active particles. EPL.

[31] Liebchen B, Löwen H (2019). Which interactions dominate in active colloids?. Chem. Phys..

[32] Stark H (2018). Artificial chemotaxis of self-phoretic active colloids: collective behavior. Acc. Chem. Res..

[33] Auschra S, Bregulla A, Kroy K, Cichos F (2021). Thermotaxis of Janus particles. Eur. Phys. J. E.

[34] Fränzl M, Muinos-Landin S, Holubec V, Cichos F (2021). Fully steerable symmetric thermoplasmonic microswimmers. ACS Nano.

[35] Selmke M, Khadka U, Bregulla AP, Cichos F, Yang H (2018). Theory for controlling individual self-propelled micro-swimmers by photon nudging I: directed transport. Phys. Chem. Chem. Phys..

[36] Selmke M, Khadka U, Bregulla AP, Cichos F, Yang H (2018). Theory for controlling individual self-propelled micro-swimmers by photon nudging II: confinement. Phys. Chem. Chem. Phys..

[37] Tunstrøm K (2013). Collective states, multistability and transitional behavior in schooling fish. PLoS Comput. Biol..

[38] Insperger, T. On the approximation of delayed systems by Taylor series expansion. *J. Comput. Nonlinear Dyn*.**10**, 024503 (2015).

[39] Muinos-Landin S, Fischer A, Holubec V, Cichos F (2021). Reinforcement learning with artificial microswimmers. Sci. Robot..

[40] Strogatz, S. H. *Nonlinear Dynamics and Chaos: With Applications to Physics, Biology, Chemistry and Engineering* (Perseus Books, 1994).

[41] Goldenfeld, N. *Lectures on Phase Transitions and the Renormalization Group* 1st edn (CRC Press, 1992).

[42] Vollmer J, Vegh AG, Lange C, Eckhardt B (2006). Vortex formation by active agents as a model for Daphnia swarming. Phys. Rev. E.

[43] Bregulla AP, Würger A, Günther K, Mertig M, Cichos F (2016). Thermo-osmotic flow in thin films. Phys. Rev. Lett..

[44] Popescu MN, Uspal WE, Dietrich S (2017). Chemically active colloids near osmotic-responsive walls with surface-chemistry gradients. J. Phys. Condens. Matter.

[45] Spagnolie SE, Lauga E (2012). Hydrodynamics of self-propulsion near a boundary: predictions and accuracy of far-field approximations. J. Fluid Mech..

[46] Lintuvuori JS, Würger A, Stratford K (2017). Hydrodynamics defines the stable swimming direction of spherical squirmers in a nematic liquid crystal. Phys. Rev. Lett..

[47] Wioland H, Woodhouse FG, Dunkel J, Goldstein RE (2016). Ferromagnetic and antiferromagnetic order in bacterial vortex lattices. Nat. Phys..

[48] Nishiguchi D, Aranson IS, Snezhko A, Sokolov A (2018). Engineering bacterial vortex lattice via direct laser lithography. Nat. Commun..

[49] Fletcher G (1997). A mechanical analog of first- and second-order phase transitions. Am. J. Phys..

[50] Kuramoto Y (1975). International symposium on mathematical problems in theoretical physics. Lect. Notes Phys..

[51] O’Keeffe KP, Hong H, Strogatz SH (2017). Oscillators that sync and swarm. Nat. Commun..

[52] Fruchart M, Hanai R, Littlewood PB, Vitelli V (2021). Non-reciprocal phase transitions. Nature.

[53] Loos SAM, Klapp SHL (2019). Fokker–planck equations for time-delayed systems via markovian embedding. J. Stat. Phys..

[54] Geiss D, Kroy K, Holubec V (2019). Brownian molecules formed by delayed harmonic interactions. New J. Phys..

[55] Drescher K, Dunkel J, Cisneros LH, Ganguly S, Goldstein RE (2011). Fluid dynamics and noise in bacterial cell-cell and cell-surface scattering. Proc. Natl. Acad. Sci. USA.

[56] Lauder GV, Drucker EG (2002). Forces, fishes, and fluids: hydrodynamic mechanisms of aquatic locomotion. Physiology.

[57] Verma S, Novati G, Koumoutsakos P (2018). Efficient collective swimming by harnessing vortices through deep reinforcement learning. Proc. Natl. Acad. Sci. USA.

[58] Morin A, Caussin J-B, Eloy C, Bartolo D (2015). Collective motion with anticipation: flocking, spinning, and swarming. Phys. Rev. E.

[59] Palmer SE, Marre O, Berry MJ, Bialek W (2015). Predictive information in a sensory population. Proc. Natl. Acad. Sci. USA.

