# Supplementary material for: Spontaneous vortex formation by microswimmers with retarded attractions
Source: Nat Commun. 2023 Jan 4;14:56. doi: 10.1038/s41467-022-35427-7 (PMC9813373; doi:10.1038/s41467-022-35427-7)
Supplement: Supplementary file 1 — Supplementary Information [file 41467_2022_35427_MOESM1_ESM.pdf]

Supplementary Information

Spontaneous vortex formation by microswimmers with retarded attractions

Xiangzun Wang[1], **Pin-Chuan Chen**[2], **Klaus Kroy**[2], **Viktor Holubec**[3] **and Frank Cichos**[1,*]

[1] *Peter Debye Institute for Soft Matter Physics, Molecular Nanophotonics Group, Universität Leipzig, 04103 Leipzig, Germany.*

[2] *Institute for Theoretical Physics, Leipzig University, Postfach 100 902, 04009 Leipzig, Germany.*

[3] *Department of Macromolecular Physics, Faculty of Mathematics and Physics, Charles Univeristy, 18000 Prague, Czech Republic*

[*] *cichos@physik.uni-leipzig.de*

Contents

1 Delay times in swarming animals **2**

2 Experimental Details **2**
2.1 Experimental setup . . . 2
2.2 Feedback system . . . 3
2.3 Active particle control . . . 3
2.4 Data analysis . . . 4
2.5 Thermophoresis and hydrodynamic flow . . . 4

3 Single particle theory **5**
3.1 Deterministic dynamics with instrumental delay and bifurcation diagram . . . 5
3.2 Stochastic dynamics: time-local approximation . . . 6
3.3 Stochastic dynamics: potential and relaxation times . . . 8
3.4 Stochastic dynamics: transition rates and effective temperatures . . . 9

4 Brownian dynamics simulations **10**

5 Collective rotation **11**
5.1 Steric interactions . . . 11
5.2 Hydrodynamic interactions . . . 12

6 Extraction of relaxation times from experimental and simulation data **15**

1 Delay times in swarming animals

Tab. S1: Mean reaction times measured between a stimulus and the corresponding discrete response strongly vary among species and the type of stimulus. Delay times comparable to the characteristic time scale of the stimulus may be expected to trigger qualitatively new effects in the dynamical response, similar to those analyzed in the present work. Specifically, bacteria (such as E. coli) are of similar size to our particles and also operate in an aqueous thermal environment.

Animal	Stimulus/Response	Reaction Time [ms]	References
Human	auditory	140 – 160	[1]
	visual	180 – 200	[1]
	touch	$\sim$ 155	[1]
Fruit fly	roll perturbation	$\sim$ 5	[2]
	pitch perturbation	$\sim$ 12	[3]
	yaw perturbation	10 – 25	[4]
Starling	startling sound stimuli	64 – 80	[5]
	startling light stimuli	38 – 76	[5]
Teleost fish	startle response	5 – 10	[6,7]
Calanoida	stirring water	< 2.5	[8]
E. coli	chemical stimuli	$\sim 10^3 - 10^4$	[9]

2 Experimental Details

2.1 Experimental setup

Fig. S1 sketches the experimental setup including the feedback system and the signal flow.

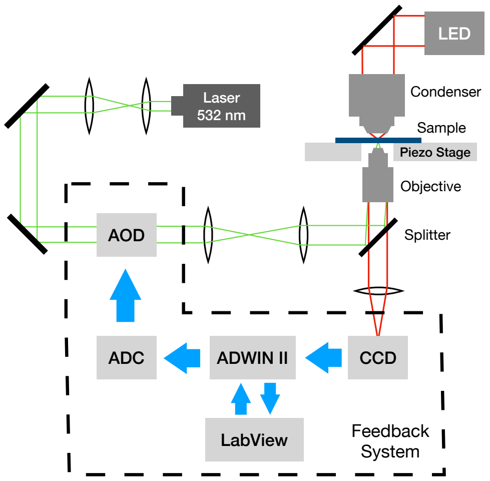

Fig. S1: Sketch of the experimental setup.

The laser of 532 nm wavelength from the module (CNI Laser, MGL-III-532) is expanded in the beam diameter by two lenses (19 mm, 100 mm focal lengths) and projected to the acousto-optic deflector (AOD, AA Opto-Electronic, DTSXY-400-532). The two perpendicularly aligned TeO_2 crystals in the AOD diffract the incident laser in horizontal and vertical directions. The deflection angle and the output laser intensity are controlled by the frequency and amplitude of the voltage on the TeO_2 which is controlled by a real-time board Adwin-Gold II (Jäger Messtechnik) including digital analog converters. The Adwin board is further controlled by a LabView program developed in the group.
Through two lenses (500 mm, 300 mm focal lengths), the laser is guided to the dark field microscope (Olympus, IX71). Reflected by a dichroic beam splitter (Omega Optical, 560DRLP), the beam is then focused by an objective (100x, Olympus, UPlanApo x 100/1.35, Oil, Iris, NA 0.5 – 1.35) on the sample. The full width of the

focus at half maximum of intensity is about 1 μm. The two lenses and the objective after the AOD convert the deflection angles to the position of laser focus on the sample. About 20 mrad range of the deflection angle corresponds to the 58×58 μm^2 area in the field of view in the microscope.
If multiple positions on the sample need to be shot by the laser, for instance to simultaneously propel multiple swimmers, the AOD keeps scanning the shoot positions circularly. Every position is exposed for 320 μs, and the position switching takes about 10 μs. The scanning order is randomized for each camera frame. Before every measurement, the AOD is calibrated with a sample of thin layer of Nile blue fluorescent dye that show the position of the laser focus on the sample. By scanning the laser in the field of view, the conversion between laser position and AOD voltage input is determined by linear interpolation.
White light from a LED lamp (Thorlabs, SOLIS-3C) is shined on the sample through an oil-immersion dark field condenser (Olympus, U-DCW, NA 1.2 – 1.4) with a glancing angle and illuminates the rims of swimmers. Hence, the swimmers can be observed as bright rings, the positions of which are evaluated in LabVIEW. The image of sample is projected through the objective and a lens (250 mm focal length) to the EMCCD camera (Andor, iXon DV885LC). The resolution of the image reaches 0.058 μm/px. A notch filter (Thorlabs NF533-17) in front of the camera blocks the residual laser reflected by the sample.

2.2 Feedback system

In the experiments, the time for signal transfer and processing in the feedback system causes an inevitable instrumental delay Δt on swimmer control. This delay is a property of the experimental setup and, unlike the delay δt in the interaction rule (see Sec. 3.1), cannot be easily controlled. Influence of both Δt and δt on the system dynamics is discussed in detail in Sec. 3.

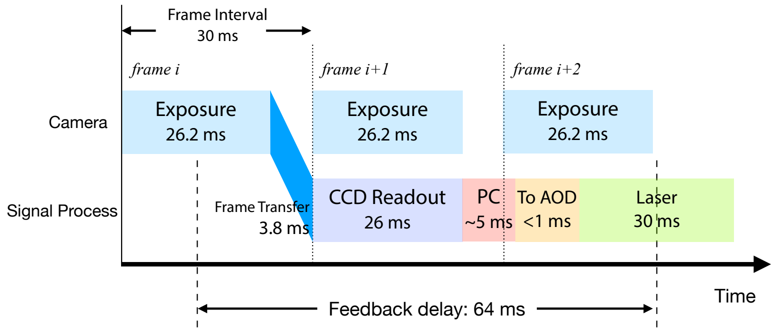

Fig. S2: Time diagram of signal processing in the feedback system for one active swimmer experiment.

Fig. S2 shows the time diagram of the signal processing in the feedback system. In the "Frame Transfer Mode", the camera is exposed while exporting the image of the previous frame. The image is read out and transferred via the Adwin-Gold II (Jäger Messtechnik) to a desktop PC (Intel Core i7 2600 4 x 3.40 GHz CPU). The LabVIEW program (v. 2019) on the PC analyzes the image and evaluates the positions of the swimmers by their bright rims. The program stores a short history of the locations of the swimmers, and tracks the swimmers by comparing their locations in previous frames. The corresponding data are recorded to a hard drive by another CPU thread. The propelling directions at time t are determined from the swimmer locations at time $t - \delta t$ in the past. The laser position for propelling is determined based on the latest measured swimmer location.
With the information from the Nile blue calibration, the laser positions are converted to voltage signals for the AOD. The signals are sent to the Adwin-Gold II, converted to analogue voltages, and transmitted to the AOD oscillator module.
The measurements with one active swimmer (Fig. 2 in the main text) used a 512×512 pixels field of view and a 30 ms frame interval. The corresponding Δt was about 64 ms. The experiments with 15 swimmers (Fig. 4 in the main text) used a larger 592×592 pixels field of view and, hence, a longer time for camera read-out resulting in a longer Δt of about 70 ms.

2.3 Active particle control

As self-propelled swimmers the experiment uses melamine formaldehyde (MF) particles of 2.19 μm diameter (microParticles GmbH) decorated with gold particles of about 0.8 nm diameter on about 10 % of their surface.

To propel the MF particle, the laser is focused at its edge at a distance of $d = 0.812$ μm from the center, as sketched in Fig. S3A.

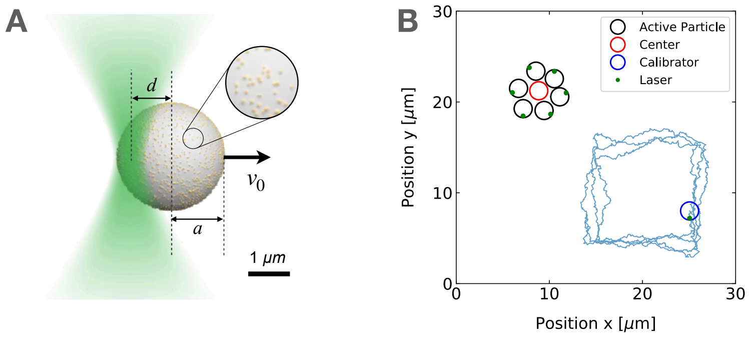

Fig. S3: (A) Sketch of the MF particle and the focused laser beam. The laser to particle distance is $d = 0.812$ μm in the experiment. Figure is reprinted from[10]. **(B)** The active swimmers (black circles), the target (red circle), and the trajectory (blue line) of the calibrator (blue circle) in a measurement.

The MF particle is transparent to the laser, which heats the gold particles and generates an asymmetric temperature gradient on the MF surface. Due to the thermophoretic effect, the swimmer is propelled away from the laser focus. For a detailed description of the propelling mechanism, see[10,11].
During the sample preparation, the swimmers can be adsorbed on the glass coverslip and become immobilized. This effect is significantly weakened by adding 0.1 % Pluronic F-127 in the sample. In the experiments, we used the remaining adsorbed swimmers as the target particles.
To keep the swimmer velocities constant during the experiment, we use a “calibrator” particle driven to patrol in the field of view far enough from the other swimmers to be independent. The calibrator is driven to sequentially change its swimming direction to follow an approximately square trajectory, as plotted in Fig. S3B. Its speed averaged over the square loop is measured in real time and the laser power is tuned by LabVIEW and the AOD to keep it constant.

2.4 Data analysis

From the recorded positions of particles in the experiments, the trajectories of the propulsion angles $\theta(t)$ between the position vectors at time t and $t - \delta t$ are determined. Note that the definition of the propulsion angle as the angle between the propulsion direction $\hat{\mathbf{u}}(t)$ and the negative position vector at time t, given on the right-hand side of Eq. (2) in the main text, is valid only for a vanishing instrumental delay Δt, and we do not use it when analyzing the data. The obtained trajectories $\theta(t)$ are used to construct the θ histograms with 10 mrad bin size shown in Fig. 2C in the main text. The velocity v_0 of the active particles is determined by the average velocity of the calibrator introduced in Section 2.3. The orbit radius R is calculated as the average distance of the active particle to the target in one measurement. For the measurements with two shells of particles (Fig. 4 in the main text), the traces with $2\ \mu\text{m} < R < 3.09\ \mu\text{m}$ are identified as particles in the inner shell, and $3.5\ \mu\text{m} < R < 6.18\ \mu\text{m}$ as those in the outer shell.

2.5 Thermophoresis and hydrodynamic flow

The swimmer is propelled due to thermophoretic effects caused by the laser-induced temperature gradient. The propulsion mechanism generates a flow of the liquid around the particle, which influences the motion of nearby particles in the measurements with multiple swimmers (e.g., see Fig. S4A). Figs. S4B and C demonstrate the hydrodynamic flow field around a fixed and a freely propelling swimmer from a COMSOL simulation. The fixed swimmer generates a flow opposite to its heading motion at the “tail”, while the moving swimmer causes the flow in the direction of its motion. These hydrodynamic and thermophoretic interactions between the particles are causing the many-body effects (co- and counter-rotating shells) depicted in Fig. 4 in the main text. Note that the swimmers in Fig. 4 are neither fully fixed nor freely movable. Depending on their propulsion angles, the corresponding flow fields are thus between those of the free and fixed swimmer.
From the trajectories of particles in a cluster, the influence of hydrodynamic flow can be deduced from the velocity distribution of the particles relative to each other (Fig. S4). The flow at the "tail" of a swimmer

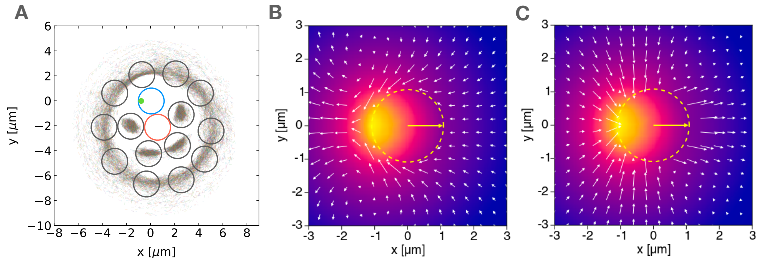

Fig. S4: (A) The distribution of 16 swimmers relative to one of them (blue circle) in a measurement. The red circle denotes the fixed target particle, and the green dot the laser. $v_0 = 2\ \mu m/s$. Panels **(B)** and **(C)** show the temperature and (induced) hydrodynamic flow fields around a fixed swimmer and a freely moving swimmer, respectively. The data were obtained using a finite element method calculation with COMSOL.

repulses other swimmers behind it. This repulsion causes the opposite rotations of different swimmer shells, as introduced in the main text (for more details, see Sec. 5). Note that Fig. S4 is calculated by the trajectories of the self-propelling active particles that obey their own rule of interaction, hence cannot be considered equivalent as the real liquid flow.

3 Single particle theory

3.1 Deterministic dynamics with instrumental delay and bifurcation diagram

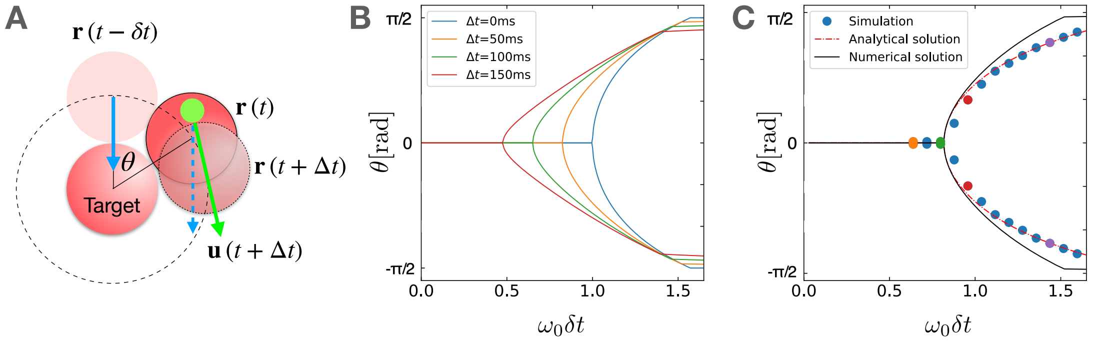

Fig. S5: (A) Sketch of the effect of the instrumental delay Δt on the motion of the swimmer. $\mathbf{r}(t)$ is the measured particle location detected by the camera, according to which the laser is focused at time $t + \Delta t$ when the position of the swimmer is $\mathbf{r}(t + \Delta t)$. **(B)** The bifurcation of the stable angle θ with different Δt's obtained numerically from Eq. (2). θ becomes constant after $\omega_0 \delta t = \pi/2$, when $\angle(\hat{\mathbf{u}}, -\hat{\mathbf{r}}) = 90^\circ$ and the swimmer leaves the target's surface. **(C)** The bifurcation diagrams obtained with $\Delta t = 64$ ms from Brownian dynamics simulations (circles), numerically (solid line), and using the approximate analytical solution (12) to Eq. (2) (dashed line). The data points with different colors correspond to the potentials in Fig. S7A. In **B** and **C**, we set $v_0 = 2.03\ \mu$m/s, $a = 1.095\ \mu$m and $d = 0.8\ \mu$m. In the simulations, we used the diffusion coefficient $D_0 = 0.0642\ \mu\text{m}^2$/s.

Consider the dynamics of a single swimmer attracted by a fixed target with the delay δt and the instrumental delay Δt, as depicted in Fig. S5A. The laser position is determined from the swimmer location $\mathbf{r}(t)$ detected by the camera at time t. However, due to the instrumental delay Δt, the actual location of the swimmer is $\mathbf{r}(t + \Delta t)$ when the laser is updated. Neglecting the noise, the swimmer displacement $\mathbf{r}(t + \Delta t) - \mathbf{r}(t)$ during Δt causes a change of its propulsion direction $\hat{\mathbf{u}} = -\hat{\mathbf{r}}(t - \delta t)$ from Eq. (1) in the main text to

$$\hat{\mathbf{u}}(t + \Delta t) = \frac{\mathbf{r}(t + \Delta t) - \mathbf{r}(t) - d\hat{\mathbf{r}}(t - \delta t)}{|\mathbf{r}(t + \Delta t) - \mathbf{r}(t) - d\hat{\mathbf{r}}(t - \delta t)|}, \tag{1}$$

where d is the programmed distance from the laser to the particle as plotted in Fig. S3A. The form $\hat{\mathbf{u}}(t) = -\hat{\mathbf{r}}(t - \delta t)$ is recovered in the limit $\Delta t \to 0$. The instrumental delay Δt makes the swimmer motion considerably

complex. It is known to amplify the effects of noise by an amount roughly proportional to $1/d$[12]. We will now show that it influences the swimmer's dynamics even if the Brownian motion is neglected.
Let us stick to the experimentally relevant situation depicted in Fig. S5 when the motile particle slides along the fixed particle and thus cannot move in the direction $-\hat{\mathbf{r}}(t+\Delta t)$. Then the true direction of motion of the motile particle is given by the projection $(1-\hat{\mathbf{r}}(t+\Delta t)\,\hat{\mathbf{r}}(t+\Delta t))$ of the propulsion direction $\hat{\mathbf{u}}(t+\Delta t)$ in Eq. (1) to the direction $(-\sin\phi(t+\Delta t), \cos\phi(t+\Delta t))$ perpendicular to $\hat{\mathbf{r}}(t+\Delta t) = (\cos\phi(t+\Delta t), \sin\phi(t+\Delta t))$. The propulsion thus creates a rotation around the fixed particle with diameter of rotation $2a$ and angular velocity $\omega(t+\Delta t) = v_0\hat{\mathbf{u}}(t+\Delta t)\cdot(-\sin\phi(t+\Delta t), \cos\phi(t+\Delta t))/(2a)$ given by

$$\frac{\omega(t)}{\omega_0} = \frac{2a\sin\theta(t,\Delta t) + d\sin\theta(t,\delta t+\Delta t)}{\sqrt{d^2+8a^2-8a^2\cos\theta(t,\Delta t)+4ad(\cos\theta(t-\Delta t,\delta t)-\cos\theta(t,\delta t+\Delta t))}}. \tag{2}$$

Here, $\theta(t,t') = \int_{t-t'}^{t} dt''\omega(t'') = \phi(t)-\phi(t-t')$ generalizes the propulsion angle from the main text. The maximum possible angular velocity of the swimmer is denoted by $\omega_0 = v_0/(2a)$. When $\Delta t = 0$, Eq. (2) simplifies to Eq. (3) in the main text.
If we assume a stable rotation with a constant angular velocity $\omega(t) = \omega \neq 0$, and neglect Brownian motion and the dependency of the swimmer velocity v_0 on the laser to the particle distance d[10], Eq. (2) can be used for generation of the bifurcation diagram. As shown in Fig. 3A in the main text, the bifurcation appears when derivatives of both sides of Eq. (2) with respect to ω at $\omega = 0$ are equal. This leads to the condition

$$\omega_0\delta t = 1 - \omega_0\Delta t\frac{2a+d}{d}. \tag{3}$$

Compared to the situation with $\Delta t = 0$, discussed in the main text, the instrumental delay shifts the critical point $\Theta_0 \equiv \omega_0\delta t$ to a lower value proportionally to $\omega_0\Delta t$. For $\Delta t \geq d/(\omega_0(2a+d))$ when the right hand side of Eq. (3) becomes negative, a stable rotation can form even with $\delta t = 0$.
The right hand side of Eq. (3) depends on ω_0, which enters the control parameter $\omega_0\delta t$. Actually, Eq. (3) can also be written as

$$\omega_0\delta t = \left(1+\frac{\Delta t}{\delta t}\frac{2a+d}{d}\right)^{-1} \equiv \Theta_B, \tag{4}$$

where the right-hand side depends on δt. These results suggest that $\omega_0\delta t$ is for $\Delta t > 0$ no longer a good control parameter and one should redefine it, e.g., by introducing the effective delay time $\delta t_{\text{eff}} = \delta t/\Theta_B$ so that the condition in Eq. (4) simplifies to $\omega_0\delta t_{\text{eff}} = 1$. Nevertheless, we keep the control parameter $\omega_0\delta t$ for sake of consistency with the main text.
The bifurcation diagram in Fig. S5B shows the numerical solutions to Eq. (2) as function of $\omega_0\delta t$ for four different values of instrumental delay Δt. In the next section, we show that the bifurcation can also be characterized analytically using a Taylor expansion. The resulting approximate formula (Eq. (12)) is compared to the numerical solution in Fig. S5C. This panel also shows the bifurcation diagram obtained from Brownian dynamics simulations of the system (for details of the simulations, see Sec. 4). The simulations reveal that the Brownian motion neglected in the above analysis causes deviations from our predictions due to fluctuations of the propulsion direction (Eq. (1)) and swimmer to target distance R. Bifurcation diagrams obtained from simulations with a much smaller diffusion coefficient than that used in the figure coincide perfectly with the numerical solution (data not shown).

3.2 Stochastic dynamics: time-local approximation

Taking into account the noise, the dynamics of the motile particle can be described by the Langevin equation[11]

$$\dot{\mathbf{r}}(t) = v_0\hat{\mathbf{u}}(t) + \mathbf{F}_{\text{int}}/\gamma_0 + \sqrt{2D_0}\boldsymbol{\eta}(t), \tag{5}$$

where $D_0 = k_{\text{B}}T/\gamma_0$ ($\sim 0.0642\ \mu\text{m}^2/\text{s}$) with friction coefficient γ_0, Boltzmann constant k_{B}, and temperature T, denotes translational diffusion coefficient, and $\boldsymbol{\eta}(t) = (\eta_x(t), \eta_y(t))^\intercal$ is a column vector of independent Gaussian white noises. The force $\mathbf{F}_{\text{int}}$ describes the hard-core interaction between the motile and fixed particle. Because the particle position $\mathbf{r}(t)$ enters the expression Eq. (1) for the propulsion direction $\hat{\mathbf{u}}(t)$ at time t with a delayed time argument, the Langevin equation is a non-linear stochastic delay differential equation[13]. In general, such equations are notoriously difficult to solve analytically, and one has to resort to approximations.

Similarly, as in the previous section, we will now assume that the distance of the motile particle from the origin is determined by twice the radius of the fixed particle, $|\mathbf{r}(t)| \sim 2a$, and we will focus only on the component of Eq. (5) that is perpendicular to the position vector $\mathbf{r}(t)$. This assumption allows us to neglect the force $\mathbf{F}_{\rm int}$ in the equation. The motion along the position vector has been investigated in a slightly different context in Ref.[11].

Projecting Eq. (5) to the direction perpendicular to $\mathbf{r}(t)$ is most easily achieved by scalar multiplying the equation by the vector $(-\sin\phi(t), \cos\phi(t))$. Using the expressions $\mathbf{r}(t) = 2a(\cos\phi(t), \sin\phi(t))$ and $\dot{\mathbf{r}}(t) = 2a(-\sin\phi(t), \cos\phi(t))\omega(t)$, where $\omega(t) = \dot{\phi}(t)$, we obtain

$$\omega(t) = \omega_0 F(t) + \sqrt{2D}\,\eta(t) \tag{6}$$

with $D \equiv D_0/(2a)^2$, and $\eta(t)$ the white noise. The factor $F(t) = \hat{\mathbf{u}}(t)\cdot(-\sin\phi(t), \cos\phi(t))$ reduces the maximum angular velocity ω_0. It is given by Eq. (2), which simplifies to $\sin\left(\int_{t-\delta t}^{t} \omega(t')\mathrm{d}t'\right)$ for a vanishing instrumental delay. Eq. (6) is still a non-linear stochastic delay differential equation, and it is virtually impossible to solve it exactly using the available techniques. However, qualitative analytical insights into the dynamics of the system can be obtained by expanding the factor F in a third-order Taylor series with respect to the delays δt and Δt. The resulting equation has the form

$$\omega(t) = c_0\omega(t) + c_1\omega^3(t) + c'\dot{\omega}(t) + c''\ddot{\omega}(t) + \sqrt{2D}\,\eta(t) \tag{7}$$

with $c' < 0$ and $c'' > 0$. This equation is local in time, and, by rearranging the terms, it can be interpreted as a Langevin equation for an underdamped Brownian particle with a mass $-c''$ and friction $-c'$ trapped in a quartic potential.

However, such a system would be unstable due to the negative mass term $-c''$, which stems from expanding the delay frequency up to second order in δt. That such higher-order expansions can lead to unstable solutions is well known in the theory of delay systems[14], and thus we set $c'' = 0$ in Eq. (7). Furthermore, the angular velocity process in Eq. (7) is already proportional to white noise $\eta(t)$, and thus the derivative $\dot{\omega}(t)$ does not exist in a strict mathematical sense. To avoid this problem, we will from now on understand $\omega(t)\delta t$ as an approximation for the propulsion angle $\theta(t) = \int_{t-\delta t}^{t} \mathrm{d}t'\omega(t')$, which works reasonably well when ω is approximately constant. Finally, we obtain the approximate time-local stochastic differential equation

$$\dot{\theta}(t) = -\left.\frac{\partial}{\partial\theta}U(\theta)\right|_{\theta=\theta(t)} + \sqrt{2D_T}\,\eta(t) \tag{8}$$

for the propulsion angle. It has the form of a Langevin equation describing a dimensionless position θ of an overdamped Brownian particle with diffusion coefficient

$$D_T = \frac{(2+\alpha)^2\Theta_B^4}{(2\Theta_B^2+\alpha)^2\Theta_0^2}\frac{D_0}{a^2} \tag{9}$$

with $\alpha \equiv d/a$, diffusing in a dimensionless quartic potential

$$U(\theta) = \frac{1}{4}f_0\theta^2(\theta^2 - 2\theta_\pm^2). \tag{10}$$

This analogy will prove immensely useful in the following discussion. The parameters in the potential read

$$f_0 = \frac{c}{(2+\alpha)(2\Theta_B^2+\alpha)\Theta_B}\frac{1}{3\delta t}, \quad \theta_\pm = \frac{(2+\alpha)\Theta_B}{\sqrt{c}}\sqrt{\frac{6}{\Theta_0}(\Theta_0 - \Theta_B)}, \tag{11}$$

where we introduced the shorthand $c = 12 - 4(3-\Theta_B)\Theta_B^2 + \alpha(6+\alpha-2\Theta_B^3)$ and, as in the main text, denoted the control parameter $\omega_0\delta t$ by Θ_0. For a vanishing instrumental delay, $\Theta_B = 1$, $c = (2+\alpha)^2$, and the potential and effective diffusion coefficient simplify to the expressions given in the main text with

$$f_0 = \frac{1}{3\delta t}, \quad \theta_\pm = \sqrt{\frac{6}{\Theta_0}(\Theta_0 - 1)}. \tag{12}$$

As stated in the main text, the quartic potential (Eq. (10)) in Eq. (8) directly maps to the Landau theory of phase transitions. Further, the dynamical equation (8) with the potential (Eq. (10)) and zero noise ($D_T = 0$) represents the normal form of the supercritical pitchfork bifurcation. Both these mappings yield expressions for the bifurcation diagram and the relaxation times. However, here we will employ a different approach based on the mentioned mapping of Eq. (8) to the overdamped diffusion, and show that, while it allows us to derive the results from the Landau theory, it also allows for characterizing the effect of the noise on the particle dynamics.

3.3 Stochastic dynamics: potential and relaxation times

The (approximate) bifurcation diagram can be deduced from the force

$$-\frac{\partial}{\partial\theta}U(\theta) = f_0\theta(\theta^2 - \theta_\pm^2). \tag{13}$$

For $\Theta_0 < \Theta_B$ it vanishes only for $\theta = 0$. On the other hand, for $\Theta_0 > \Theta_B$ the force vanishes for $\theta = 0$ and $\theta = \pm\theta_\pm$. The bifurcation diagram shows the stationary solutions to the differential equation which thus must be stable to small perturbations. We introduce two ways how to determine the stability.
The simpler one is based on the potential: stable stationary solutions correspond to its local minima and unstable solutions to its local maxima. The potential $U(\theta)$ as function of the characteristic parameter $\Theta_0 = \omega_0\delta t$ is depicted in Fig. S6. From the figure, it follows that $\theta = 0$ is a stable solution only for $\Theta < \Theta_B$, where it corresponds to a single minimum of the potential. For $\Theta > \Theta_B$, the potential develops two local minima and the system allows for two symmetric stable solutions $\theta = \pm\theta_\pm$. In accord with the discussion from the preceding section, the bifurcation appears at $\Theta = \Theta_B$ when a single stable solution changes to two stable solutions.

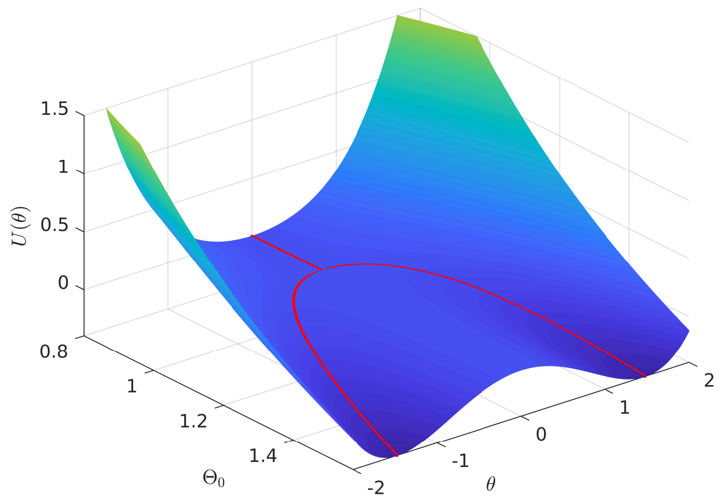

Fig. S6: The potential $U(\theta)$ as function of the control parameter $\Theta_0 \equiv \omega_0\delta t$ according to Eq. (10) with $\Delta t = 0$, corresponding to $\Theta_B = 1$ according to Eq. (4). With increasing Θ_0, $U(\theta)$ transforms from a single well to a double well potential. The red curve denotes the local minima of $U(\theta)$ and thus the stable angles $\theta_\pm$ in the bifurcation diagram in Figs. S5B and C.

Another way to identify the stable solutions, which, moreover, yields approximate relaxation times of small perturbations from the stable points, is the linear response theory. Let us first investigate the stability of the solution $\theta = 0$. Assuming small perturbations around this point, i.e., taking $\theta = \delta\theta$ in Eq. (8) and expanding the result up to the first order in $\delta\theta$, we find the equation

$$\frac{d}{dt}\delta\theta(t) = f_0\theta_\pm^2\delta\theta(t), \tag{14}$$

which describes an exponential relaxation $\delta\theta \propto \exp(-t/\tau)$ with the relaxation time

$$\tau = -\frac{1}{f_0\theta_\pm^2} = \frac{2\Theta_B^2 + \alpha}{2+\alpha}\frac{\delta t}{2\left(\frac{1}{\Theta_0} - \frac{1}{\Theta_B}\right)}. \tag{15}$$

This relaxation time is positive for $\Theta_0 < \Theta_B$ and negative for $\Theta_0 > \Theta_B$. At the bifurcation, $\Theta_0 \to \Theta_B$, the relaxation time diverges, which is the manifestation of critical slowing down. Stationary solutions are stable if the corresponding relaxation time is positive and thus the solution $\theta = 0$ is stable for $\Theta_0 < \Theta_B$.
Repeating this procedure for small perturbations around $\theta = \pm\theta_+$, we find that the corresponding relaxation time is given by

$$\tau = -\frac{2\Theta_B^2 + \alpha}{2+\alpha}\frac{\delta t}{4\left(\frac{1}{\Theta_0} - \frac{1}{\Theta_B}\right)}. \tag{16}$$

This relaxation time is negative for $\Theta_0 < \Theta_B$, positive for $\Theta_0 > \Theta_B$, and diverges for $\Theta_0 \to \Theta_B$. The solutions $\theta = \pm\theta_+$ are thus stable for $\Theta_0 > \Theta_B$. The linear stability analysis hence leads to the same bifurcation diagram as predicted from the potential in Fig. S6. The positive relaxation times constitute the system discussed in the main text. For vanishing instrumental delay $\Delta t = 0$, the formulas (15) and (16) simplify to Eq. (7) in the main text.
The theoretical predictions (15) and (16) are compared to relaxation times obtained from experiments and Brownian dynamics simulations in Fig. S11C in Sec. 6, where we discuss two complementary methods for extracting the relaxation times from the data.

3.4 Stochastic dynamics: transition rates and effective temperatures

In the regime when the potential becomes bi-stable, the noise induces transitions between the two minima. Employing the overdamped interpretation of Eq. (8), it seems natural to use the Kramers' rate theory[15] to describe these transitions. For an overdamped Brownian particle diffusing in a double well potential U with minima at $\pm\theta_\pm$, the Kramers theory predicts that transitions between the two wells appear with the transition rate

$$k = \frac{1}{2\pi}\sqrt{-U''(0)U''(\theta_\pm)}\exp\left(\frac{-E_b}{D_\theta}\right). \tag{17}$$

Here, $E_b = U(0) - U(\theta_\pm)$ is the height of the energetic barrier between the minima, and D_θ denotes the thermal energy, which is, in our units with unit friction coefficient, given by the diffusion coefficient. Using the potential and the diffusion coefficient from Eq. (8) thus leads to the prediction

$$k = \frac{f_0\theta_\pm^2}{\sqrt{2}\pi}\exp\left(-\frac{f_0\theta_\pm^4}{4D_\theta}\right), \tag{18}$$

with $D_\theta = D_T$. The red solid line and the circles in Fig. S7C show that this prediction unfortunately does not agree with transition rates evaluated from Brownian dynamics simulations of Eq. (5). This disagreement is caused by the uncontrolled approximations used in our derivation. Actually, there is no guarantee that a delay stochastic differential equation can be approximated by a time-local stochastic differential equation. The only case where such a mapping can be derived rigorously are linear stochastic differential equations, which can be shown to be equivalent to time-local stochastic differential equations with highly nontrivial time-dependent coefficients and a colored noise[13]. But even there the transition rates differ from those predicted using the simple overdamped description[13].
In the present situation, the agreement of the theoretically predicted bifurcation diagram and relaxation times with the experimental and simulation data shown in Figs. S5 and S11, and Fig. 3 in the main text gives us confidence that the potential Eq. (10) approximates well the systematic part of the delay force in Eq. (5). To improve the prediction for the transition rate, we thus decided to fit the diffusion coefficient D_θ in Eq. (18) to the data. However, to further test the validity of the suggested mapping of Eq. (5) to the time-local overdamped Eq. (8), we have not determined D_θ by fitting the transition rates. Instead, we fit the Boltzmann distribution

$$p(\theta) = \frac{\exp(-U(\theta)/(D_\theta))}{\int_{-\infty}^{\infty}\exp(-U(\theta)/(D_\theta))} \tag{19}$$

to the distributions for θ determined from experiments and simulations. Examples of such fits to $-\log p(\theta)$ obtained from simulations and experiments are depicted in Fig. S7A and Fig. 2C in the main text, respectively. The corresponding diffusion coefficients D_θ are shown in Fig. S7B together with the prediction (9). Both D_θ obtained from experiments and simulations converge to (different) constant values depicted in the figure by horizontal lines. The large fluctuations in D_θ obtained from experimental data for small values of $\omega_0\delta t$, shown in the inset, are caused by insufficient statistics around the central peak of $-\log p(\theta)$, rendering the fitting results unreliable.
The transition rates calculated using Eq. (18) with the fitted D_θ from Fig. S7B are depicted by symbols in Fig. S7C for the simulations and Fig. 3D in the main text for the experiments. Besides using the fitted $\omega_0\delta t$ dependent diffusion coefficient, we also plotted the transition rates using the constant plateau values $D_\theta = 0.034$ s^{-1} and $D_\theta = 0.05$, respectively for the simulation and the experiment. The remarkable agreement of the resulting transition rates with rates directly measured from simulation and experiments reveals that, at least

from the perspective of transition rates, the delay stochastic differential Eq. (5) can be well approximated by the time-local overdamped Langevin Eq. (8) with the potential Eq. (10) and white noise with diffusion coefficient D_θ instead of D_T from Eq.(9).

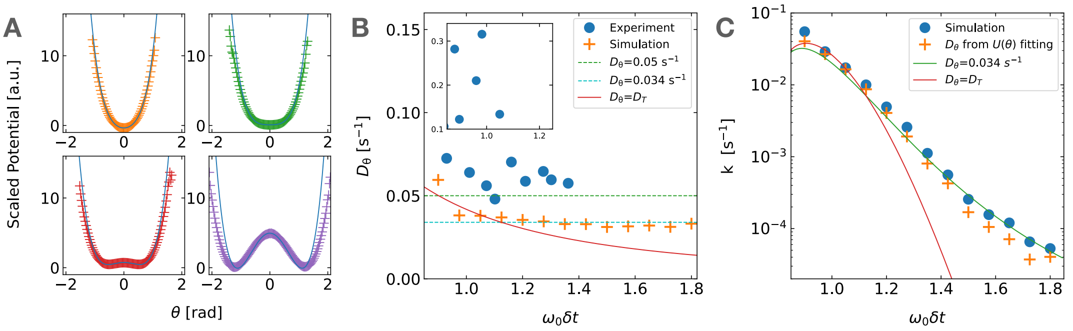

Fig. S7: (A) Fits of the Boltzmann distribution (Eq. (19)) with the potential (Eq. (10)) (lines) to $-\log p(\theta)$ from simulations ($+$ symbols) for $\omega_0\delta t = 0.60, 0.75, 0.90,$ and 1.35, denoted by the same colors as the corresponding data points in Fig. S5C. **(B)** The diffusion coefficient D_θ determined by the fitting of experimental and simulated distributions for θ together with the theoretical prediction (Eq. (9)). **(C)** Transition rates k for jumps of θ between the two wells of the potential, measured from the simulations (circles) and the predictions from Kramers' theory (Eq. (18)) with different diffusion coefficients D_θ. The other parameters are the same as in Fig. S5C.

4 Brownian dynamics simulations

To test the approximate theoretical results beyond the parameter regimes accessible in experiments, we simulated the non-approximate Langevin equation (5) with the timestep $\mathrm{d}t = 10$ ms. To take into account finite sizes of the motile and the fixed particle, we assumed that $\mathbf{F}_{\mathrm{int}}$ describes a hard core repulsion, which restricts $\mathbf{r}(t)$ to $(2a, \infty)$.

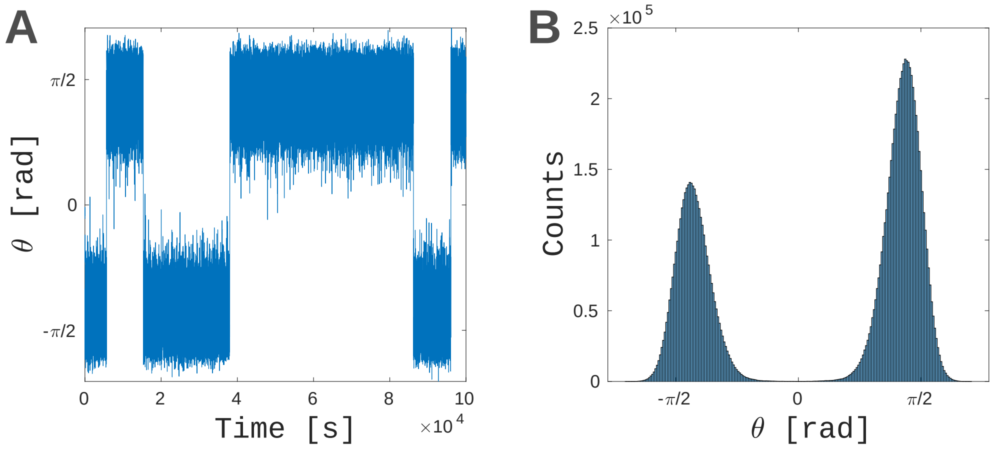

Fig. S8: Sample data from Brownian dynamics simulation for $v_0 = 2.16$ μm/s and $\delta t = 1.62$ s. **(A)** The trajectory of the angle $\theta(t)$ fluctuates around the stable values $\theta_\pm$. **(B)** The corresponding histogram for θ. The other parameters are the same as in Fig. S5C.

Most of our simulations take into account all key ingredients of the experiment except for hydrodynamic interactions. Therefore, the simulated data show excellent agreement with single particle measurements, cf the example simulated trajectory of the delay angle and the corresponding histogram shown in Fig. S8A (Fig. S8B) to the experimental data in Fig. 2A (Fig. 2C) of the main text. However, our simulations of many-body systems exhibit significant disagreement from data from multiple particle experiments, especially if the particles form

several co- or counter-rotating layers. This disagreement allows us to conclude that the effects observed in many-body experiments, like the shift of the bifurcation to smaller values of control parameter and counter-rotating particle layers, are solely induced by hydrodynamic interactions among the active particles. As shown in Fig. S9 and discussed in the Sec. 5.1, the only significant many-body effect that is present in the simulations without hydrodynamic interactions is stabilization of the system (decrease of transition rate between clockwise and counter-clockwise rotation) with increasing number of particles.
We have adapted phenomenological backflow-induced forces in some simulations to study the role of hydrodynamic (and phoretic) interactions without reproducing the whole experiment in silico. We constructed them using three leading terms (monopole, dipole, and polar contributions) in the expansions of the hydrodynamic velocity field from Refs.[16] and[17]. We switched the monopole field decaying as (1/distance) only when the target particle blocked the propelled particle. In the formula for the force exerted by the backflow, we additionally neglected the "diffusive" term proportional to the second derivative of the velocity field. We have chosen the coefficients in the three contributions so that the phenomenology observed in simulations and experiments agrees. In this sense, our simulation can also phenomenologically incorporate some phoretic effects described by the same decay laws as the considered hydrodynamic contributions. In Fig. S10 and Sec. 5.2 below, we demonstrate that these simulations reproduce the co- and counter-rotating shells observed in experiments.

5 Collective rotation

As described in the main text, considering an ensemble of particles each of which is driven towards the same target by the same retarded interaction leads to interesting collective effects. Due to steric, hydrodynamic and thermophoretic interactions, the particles rotate around the target in shells. In each of the shells, the inter-particle interactions synchronize and stabilize the rotation of the particles, as shown in Fig. S4A. The transition rate for switching between clockwise and counter-clockwise rotation thus decreases with the number of particles in a shell. Otherwise, the bifurcation in a given shell occurs approximately for the same parameters as for a single particle. The inter-particle interactions also couple dynamics of the neighbouring shells, which can either stably co-rotate or counter-rotate.

5.1 Steric interactions

To understand the effect of steric interactions alone in the multi-particle rotation, we performed BD simulations without hydrodynamic forces and instrumental delay for N ranging from 1 to 69. For $N = 6$ and $\delta t < \pi a/v_0$, the swimming particles form a closed hexagonal shell around the target particle of radius $R \simeq 2a$. For larger delays, the shell is no longer closely packed as its diameter increases to $R \sim 2v_0\delta t/\pi$. For $N > 6$, the innermost shell is pressed towards the central particle and its diameter returns to $R \simeq 2a$. The other shells form an optimal hexagonal lattice with $6k$ particles in the kth shell. Assuming that the particles eventually fill a circle, the radius of the outermost shell can be estimated as $a\sqrt{N/0.907}$, where 0.907 is the density of the optimal circular packing in two dimensions.
Up to $N = 6$ particles, the steric interactions just synchronize and stabilize the rotation. The bifurcation diagrams for $1 \leq N \leq 6$ in Fig. S9A thus collapse to a single curve described by the single-particle theory (Eq. (5) in the main text). In Fig. S9B, we show that the corresponding bifurcation diagrams obtained from the experiments also approximately collapse for $N \leq 6$. We attribute the discrepancies in the collapse to hydrodynamic and phoretic interactions, neglected in the simulation, and measurement uncertainties. More detailed studies are subject of future work.
The stabilizing effect of the steric inter-particle interactions is best visualized by the exponential decrease of the transition rate for jumps between the two rotating states depicted in Fig. S9C. This linear scaling of the exponent in the transition rate with N can be intuitively understood as follows. Before the whole set of particles in a given shell changes its sense of rotation, it forms a "train of compartments" which can be thought of as a single quasiparticle with an effective diffusion coefficient that scales as $1/N$ due to the central limit theorem. As the exponent in the transition rate is proportional to the inverse of the diffusion coefficient, this effect yields the observed linear scaling of the logarithm of the transition rate.
Finally, in Fig. S9D, we show bifurcation diagrams for $N \geq 14$ obtained from simulations without hydrodynamic interactions by averaging the propulsion angles of all particles in the ensemble. After rescaling the control parameter $v_0\delta t$ by the radius $R \sim a\sqrt{N/0.907}$ of the outermost shell and a constant factor of 1.33, all these diagrams fall onto a single master curve described by the single-particle theory. For short delays, all particles

move with the same angular velocity as the outer shells propel toward the center of the flock, thus effectively imposing their angular velocity on the inner shells. With the increasing delay, the propulsion direction of the particles becomes more and more tangential, and the strength of the blocking drops. The outer shells lack behind the inner ones as the maximum angular velocity of the particles $v_0\delta t/R$ decreases with the distance from the center. In both these regimes, the particles thus move on average with a smaller angular velocity than a single particle. It turns out that this "slowing down" is nicely described by the constant $1.33 \approx 4/3$ we used to upscale the speed in the bifurcation diagram. Unfortunately, we cannot compare these simulation results to experimental data as control of such large ensembles of particles is beyond our experimental setup's current capabilities.

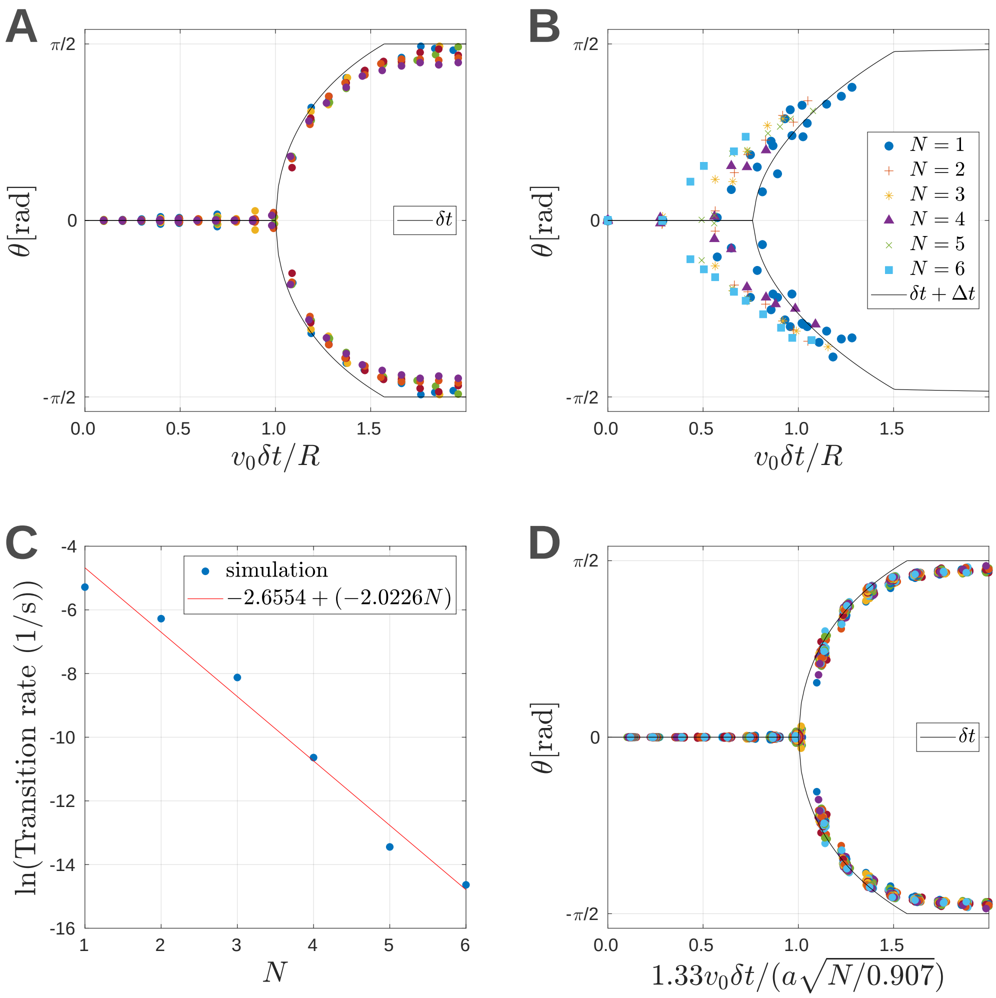

Fig. S9: Collective behavior in simulations with only steric interactions and experiments. **(A)** The bifurcation diagrams for $1 \leq N \leq 6$ obtained from simulations. The data collapse on the solid line corresponding to the single particle theory (Eq. (5) in the main text). **(B)** The bifurcation diagram for $1 \leq N \leq 6$ extracted from experimental data. The solid line is the theoretical prediction (11) for the bifurcation curve that includes both the programmed delay δt and the instrumental delay Δt. In (A) and (B), $R \approx 2a$ denotes the average distance of the particles from the center. **(C)** The logarithm of the transition rate for switching between clockwise and counter-clockwise rotation for $1 \leq N \leq 6$, obtained from simulations. **(D)** The bifurcation diagram for $14 \leq N \leq 69$ obtained from simulations. After rescaling, the data collapse on the solid line corresponding to the single particle prediction.

5.2 Hydrodynamic interactions

now focus in detail on the the behavior in the two-shell scenario depicted in Fig. 4 in the main text. The maximum angular velocity in the inner shell, $\omega^{in} = v_0/(2a)$, is two times larger than that in the outer shell,

$\omega^{out} = v_0/(4a)$. Therefore, one might conclude that the inner shell can rotate and the outer shell moves randomly. However, rotation in the inner shell always induces at least a weak rotation in the outer shell. As the control parameter $\omega^{out}\delta t$ increases, the outer and inner shells start to counter-rotate. Finally, an even stronger increase in $\omega^{out}\delta t$ leads to co-rotation of the two shells. This complicated behavior, caused by the hydrodynamic and thermophoretic interactions between particles in the two layers, can be understood using a simple phenomenological model. Let the particles in the inner shell stably rotate with a propulsion angle θ^{in}. Then the dynamics of the propulsion angle θ^{out} of particles in the outer shell can be qualitatively described by the formula

$$\theta^{out}(t) = \omega^{out}\delta t\left[\sin\theta^{\mathrm{out}}(t-\delta t) - B\sin\theta^{\mathrm{in}}\cos\theta^{\mathrm{in}} + C\sin\theta^{\mathrm{in}}\right] + \sqrt{2D_0/R^2}\xi(t). \tag{20}$$

The first term on the right-hand side comes from the delayed attraction to the center and leads to the single-particle potential in Eqs. (10) and (12) and the bifurcation diagram in Fig. (S5). The second term has the opposite sign than θ^{in} and thus causes the counter-rotation of the two shells. The third term has the same sign as θ^{in} and causes the co-rotation. The last term stands for the thermal noise. The dependency of the second and third terms on θ^{in} can be understood as follows.

Addressing a particle by the laser causes flows of water which induce swimming of the particle. These flows are strongest in the direction opposite to the particle motion. When a particle is propelled with delay angle θ^{in}, the backflow is thus strongest in the direction $(-\sin\theta^{in}, \cos\theta^{in})$ in the coordinate system defined by the vector parallel to the particle's circular trajectory, and the position vector $\mathbf{r}(t)$, see Fig. S5A. If the backflow from the inner shell penetrates into the outer shell, it can cause the co- and counter-rotations (see Fig. 4 in the main text). The co-rotation can be induced if the delay angle θ^{in} is so large that the backflow hits the particle in the inner shell and is reflected into the outer shell with opposite direction. We assume that this effect can be modeled by the term proportional to $\sin\theta^{in}$, i.e., to the component of the backflow tangential to the inner layer. The counter rotation is then induced by the amount of backflow which penetrates into the second layer. This portion of the backflow is proportional to $\cos\theta^{in}$. However, the leaked backflow can only cause rotation if it has a nonzero component within the second layer. This component is again proportional to $\sin\theta^{in}$. Altogether, we assume that the counter-rotation is caused by the term proportional to $\sin\theta^{in}\cos\theta^{in}$. Finally, we assume that the proportionality factor $C > 0$ for the reflected backflow is smaller than $B > 0$, describing the direct effect of the backflow.

The phenomenological bias $\omega^{out}\delta t(-B\sin\theta^{in}\cos\theta^{in} + C\sin\theta^{in})$ from the inner shell tilts the potential (10) and thus stabilizes one of the rotating states. For $\Theta_0 < 1$, there is no rotation in the inner shell, and thus $\theta^{in} = 0$ and the bias vanishes. For $\Theta_0 > 1$, θ^{in} is in the present approximation given by Eq. (12). For $0 < \theta^{in} < \arccos(C/B)$, the term $-B\sin\theta^{in}\cos\theta^{in}$ dominates and the bias favors θ^{out} with opposite sign than θ^{in}, and the other way round for $\arccos(C/B) < \theta^{\mathrm{in}}$. Inserting $\theta^{in} = \sqrt{6(1-1/\Theta_0)}$ from Eq. (12) in the condition $\theta^{in} = \arccos(C/B)$, we find that the transition from counter- to co-rotation occurs for

$$\Theta_0 = \frac{6}{6 - \arccos^2(C/B)}. \tag{21}$$

Depending on the value of the ratio of the phenomenological parameters B and C, the expression on the right-hand side interpolates between the values $-24/(\pi^2 - 24) \sim 1.7$ for $C/B = 0$ and 1 for $C/B = 1$. Translating this result to the language of time-delay δt, velocity v_0, and radius $2a$ of the inner-shell, we find

$$v_0\delta t = 12a/[6 - \arccos^2(C/B)]. \tag{22}$$

In Fig. S10B, we show the phase diagram for co and counter-rotating phases obtained from the simulation with steric repulsion, phenomenological hydrodynamic forces, and the instrumental delay of $\Delta t = 0.064$ s (see Sec. 4 for details). The corresponding diagram obtained from experiments is shown in Fig. S10A. Eq. (22) describes the phase boundary in both these diagrams. However, the corresponding values of B/C for the simulation ($B/C \sim 0.2$) and experiments ($B/C \sim 1.0$) are much different. We attribute this discrepancy to the rough implementation of hydrodynamic and phoretic interactions in the simulation and the analytical model. The analytical model further does not incorporate steric interactions, which play an important role in the observed synchronization. Noteworthy, all these different implementations of the backflow yield the same phenomenology, which points to its robustness against details of the interactions' shape.

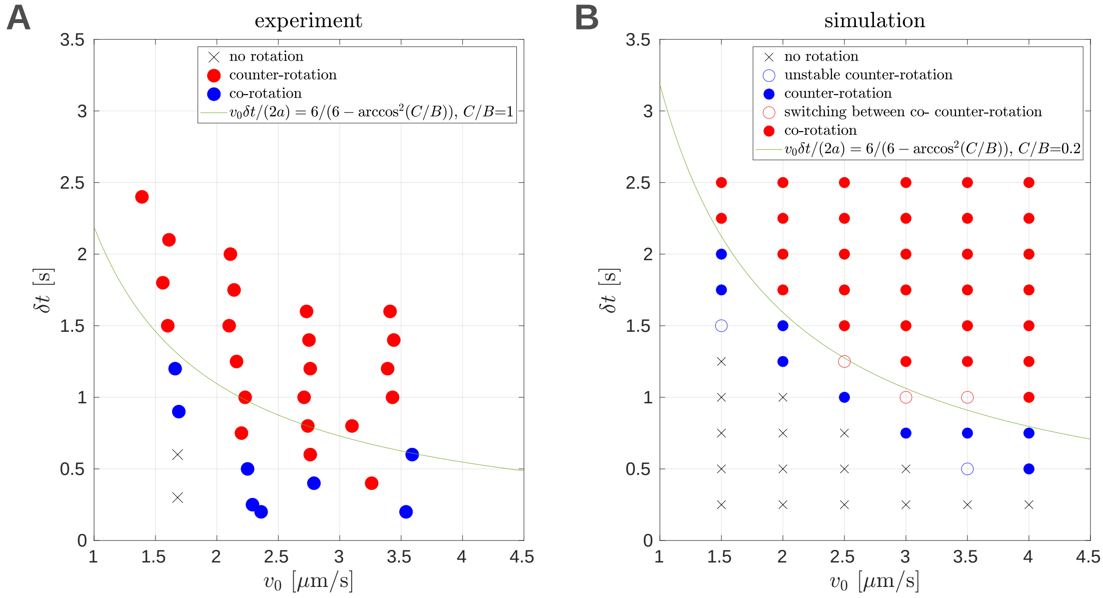

Fig. S10: Phase diagram of counter/co-rotating shells for $N = 15$ obtained from experiments (A) and simulations (B). The simulations include the instrumental delay $\Delta t = 0.064$ s and effective hydrodynamic interactions. The phase boundaries are described by Eq. (22) with $C/B \simeq 1$ for experiments and 0.2 for simulations, corresponding to $v_0\delta t/(2a) = 1$ and $v_0\delta t/(2a) = 1.45$, respectively. In addition to the two rotational phases observed in the experiments, we observed unstable/switching modes on their boundary in simulations.

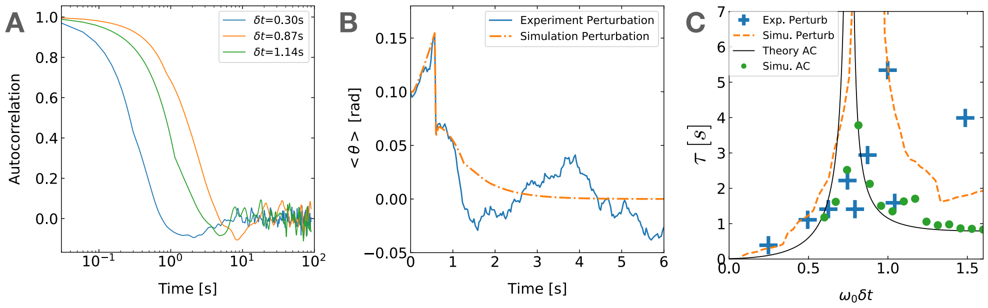

Fig. S11: (A) Auto-correlation (AC) function of $\theta(t)$ corresponding to the three colored experimental data points in Fig. 2 of the main text. **(B)** An example of the relaxation of average $\theta(t)$ after a 0.1 rad perturbation of the stable non-rotating state. The solid line was measured in experiments and the dashed line was obtained numerically from Eq. (5). **(C)** The relaxation time τ from theory (Eqs. (15) and (16), solid line), AC functions determined from Brownian dynamics simulations (circles), and relaxation of the perturbation exemplified in **(B)** in experiments (crosses), and numerics (dashed line). We used $v_0 = 1.7$ μm/s **(B)**, $\delta t \approx 0.6$ s (**(B)** and **(C)**), $a = 1.095$ μm and $d = 0.8$ μm.

6 Extraction of relaxation times from experimental and simulation data

There are two complementary ways to determine the relaxation times in the two stable states from experimental and simulation data. The first one is closely related to the idea of the linear stability analysis: one induces a small perturbation of the system from its stable state and measures how long it takes for this excitation to decay to $1/e$ its initial value. If one cannot easily induce such a perturbation in the system, it is still possible to obtain the relaxation time as the decay time of the stationary time-correlation function calculated from trajectories trapped in the stable state.

The complementarity of the two approaches is based on the linearized Langevin equation

$$\frac{d}{dt}\delta\theta(t) = -\frac{1}{\tau}\delta\theta(t) + \eta(t) \tag{23}$$

approximately describing time evolution of small perturbations $\delta\theta(t) = \theta(t) - \theta_S$ of a stable state θ_S. Both the average solution to this equation, $\langle\delta\theta(t)\rangle$ and its stationary normalized auto-correlation function,

$$C(t) = \frac{\langle\delta\theta(t+t')\delta\theta(t')\rangle_{t'}}{\langle\delta\theta(t')^2\rangle_{t'}} \equiv \lim_{t_m\to\infty}\frac{\int_0^{t_m}\mathrm{d}t\,\delta\theta(t+t')\delta\theta(t')}{\int_0^{t_m}\mathrm{d}t\,(\delta\theta(t'))^2}, \tag{24}$$

decay exponentially with the relaxation time τ. In the definition of the time correlation function, t_m denotes the final time of the measurement and it is assumed that the process $\theta(t)$ is initialized in a distant time in the past, $t_i \to -\infty$, so that it is already stationary at $t = 0$.

For an overdamped system with a harmonic potential, the two methods yield exactly the same relaxation time. For a nonlinear delay stochastic differential equation such as Eq. (5), the correspondence is not necessarily exact. However, also linearized stochastic delay differential equations yield average solutions with the same decay rate as the stationary correlation functions[13]. The two approaches can thus be expected to yield the same results whenever the system evolves most of the time close to the minimum of the potential, which is well approximated by a parabola. Such conditions are achieved whenever the thermal energy $k_\mathrm{B}T$ is small compared to a characteristic energy scale of the potential. That the two approaches yield similar results also in our situation is demonstrated in Fig. S11C and in Fig. 3C in the main text, where we compared the theoretical results Eqs. (15) and (16) and relaxation times τ obtained from experiments and simulations using the two methods described above. More details are given in the following two paragraphs.

The time correlation functions Eq. (24) determined from experimental trajectories of $\theta(t)$ trapped in the individual stable states ($\theta(t)$ in the ± 1 rad range of the theoretical values 0 or $\theta_\pm$) are shown in Fig. S11A. The corresponding decay times were determined as times needed for the correlation function to decay to $1/e$ of its initial value. The resulting decay times averaged over data obtained from all trajectories trapped in a given state are shown in Fig. 3C in the main text. Similar time correlation functions also follow from the Brownian dynamics simulations. The corresponding decay times are depicted by solid circles in Fig. S11C. These decays times were evaluated in the same way as in experiments with the difference that we employed the formula

$$\int_0^\infty \mathrm{d}t\,\sin\left(\frac{t}{\tau}\right)\frac{C(t)}{t} = \frac{\pi}{4} \tag{25}$$

designed to extract the most relevant timescale of a decaying function[18] to extract the decay time from the individual correlation functions. Both the experimental and the simulation results are in good agreement with the theoretical predictions Eqs. (15) and (16) according to the linear stability analysis (solid lines). We also verified that the simulation data agree with theoretical predictions better for smaller temperatures (data not shown).

An example of the time evolution of a perturbation $\delta\theta = 0.1$ applied to the stationary non-rotating state $\theta_S = 0$ at $t = 0$, corresponding to $\omega(t) = \delta\theta(t)$ proportional to the δ-function for $t \le 0$, is shown in Fig. S11B. The dashed line results from numerical integration of Eq. (5) averaged over the noise. The solid line was measured in the experiment, where we averaged over 150 perturbation-relaxation cycles. During the time interval $(0, \delta t)$, the average propulsion angle $\theta(t) = \int_{t-\delta t}^{t}\mathrm{d}t'\,\omega(t')$ is given by the perturbation and increases as $\omega(t) = \omega_0 \sin\theta(t)$ is positive. At time $t = \delta t$, $\theta(t)$ suddenly drops by $\delta\theta$ as the perturbation no longer influences the angular velocity. This change then causes a next drop at $t = 2\delta t$ etc. Such a non-smooth time evolution is for delay differential equations typical[13].

For rotating stable states, the decay of a finite perturbation depends on its sign due to a slight asymmetry of the corresponding effective potential. Therefore, we evaluated the relaxation trajectories using the same

way as for $\theta_S = 0$ but we in addition averaged over the perturbations $\delta\theta = \pm 0.1$. The decay times τ of the resulting averaged relaxation trajectories, determined as $\theta(\tau) = \theta(0)/e$, are plotted as dashed lines (numerics) and crosses (experiments) in Fig. S11C. The non-smoothness of the resulting functions is related to the non-smooth evolution of the relaxation trajectories. The large fluctuations of the experimental results close to the bifurcation point, where the relaxation time diverges, arise from finite measurement time (6s) per relaxation event.

References

[1] R. J. Kosinski, A literature review on reaction time, *Clemson University* **10**, 337–344 (2008).

[2] T. Beatus, J. M. Guckenheimer, and I. Cohen, Controlling roll perturbations in fruit flies, *Journal of The Royal Society Interface* **12**, 20150075 (2015).

[3] L. Ristroph, G. Ristroph, S. Morozova, A. J. Bergou, S. Chang *et al.*, Active and passive stabilization of body pitch in insect flight, *Journal of The Royal Society Interface* **10**, 20130237 (2013).

[4] L. Ristroph, A. J. Bergou, G. Ristroph, K. Coumes, G. J. Berman *et al.*, Discovering the flight autostabilizer of fruit flies by inducing aerial stumbles, *Proceedings of the National Academy of Sciences* **107**, 4820–4824 (2010).

[5] H. Pomeroy and F. Heppner, Laboratory determination of startle reaction time of the starling (Sturnus vulgaris), *Animal Behaviour* **25**, 720–725 (1977).

[6] R. C. Eaton, R. A. Bombardieri, and D. L. Meyer, The Mauthner-initiated startle response in teleost fish, *Journal of Experimental Biology* **66**, 65–81 (1977).

[7] R. C. Eaton, *Neural mechanisms of startle behavior* (Springer Science & Business Media, 1984).

[8] P. Lenz and D. Hartline, Reaction times and force production during escape behavior of a calanoid copepod, Undinula vulgaris, *Marine Biology* **133**, 249–258 (1999).

[9] J. E. Segall, S. M. Block, and H. C. Berg, Temporal comparisons in bacterial chemotaxis, *Proceedings of the National Academy of Sciences of the United States of America* **83**, 3024160[pmid], 8987–8991 (1986).

[10] M. Fränzl, S. Muiños-Landin, V. Holubec, and F. Cichos, Fully Steerable Symmetric Thermoplasmonic Microswimmers, *ACS Nano* **15**, 3434–3440 (2021).

[11] U. Khadka, V. Holubec, H. Yang, and F. Cichos, Active Particles Bound by Information Flows, *Nat. Commun.* **9**, 3864 (2018).

[12] S. Muiños-Landin, A. Fischer, V. Holubec, and F. Cichos, Reinforcement learning with artificial microswimmers, *Science Robotics* **6** (2021).

[13] D. Geiss, K. Kroy, and V. Holubec, Brownian molecules formed by delayed harmonic interactions, *New Journal of Physics* **21**, 093014 (2019).

[14] T. Insperger, On the Approximation of Delayed Systems by Taylor Series Expansion, *Journal of Computational and Nonlinear Dynamics* **10**, 024503 (2015).

[15] P. Hänggi, P. Talkner, and M. Borkovec, Reaction-rate theory: fifty years after Kramers, *Rev. Mod. Phys.* **62**, 251–341 (1990).

[16] I. Llopis and I. Pagonabarraga, Hydrodynamic interactions in squirmer motion: Swimming with a neighbour and close to a wall, en, *Journal of Non-Newtonian Fluid Mechanics* **165**, 946–952 (2010).

[17] A. I. Campbell, S. J. Ebbens, P. Illien, and R. Golestanian, Experimental observation of flow fields around active Janus spheres, en, *Nature Communications* **10**, 3952 (2019).

[18] B. I. Halperin and P. C. Hohenberg, Scaling Laws for Dynamic Critical Phenomena, *Phys. Rev.* **177**, 952–971 (1969).
