# Supplementary material for: Spontaneous vortex formation by microswimmers with retarded attractions
Source: Nat Commun. 2023 Jan 4;14:56. doi: 10.1038/s41467-022-35427-7 (PMC9813373; doi:10.1038/s41467-022-35427-7)
Supplement: Supplementary file 3 — Description of Additional Supplementary Files [file 41467_2022_35427_MOESM3_ESM.pdf]

File Name: Supplementary Movie 1:

Description: Video recorded in real-time experiment, demonstrating single active particle attracted to a fixed particle. Delay $\delta t = 0.3$ s as in Fig. 2A of the main text. The bright circles denote the particles in the dark-field microscope. The particle at the right top corner is the calibrator to determine the speed v_0.

File Name: Supplementary Movie 2:

Description: Video from real-time experiment of single active particle attracted to a fixed particle. Delay $\delta t = 0.87$ s as in Fig. 2B of the main text.

File Name: Supplementary Movie 3:

Description: Video from real-time experiment of single active particle attracted to a fixed particle. Delay $\delta t = 1.14$ s as in Fig. 2C of the main text.

File Name: Supplementary Movie 4:

Description: Video from real-time experiment of 15 active particles attracted to a fixed target particle. Delay $\delta t = 0.8$ s as in Fig. 4C of the main text. The rotating inner shell and non-rotating outer shell of the active particles can be observed.

File Name: Supplementary Movie 5:

Description: Video from real-time experiment of 15 active particles attracted to a fixed particle. Delay $\delta t = 1.35$ s as in Fig. 4C of the main text. The counter-rotations of the inner and outer shells of particles can be observed.

File Name: Supplementary Movie 6:

Description: Video from real-time experiment of 15 active particles attracted to a fixed particle. Delay $\delta t = 1.65$ s as in Fig. 4C of the main text. The co-rotation of the inner and outer shells of particles can be observed.
