# Supplementary material for: Spontaneous vortex formation by microswimmers with retarded attractions
Source: Nat Commun. 2023 Jan 4;14:56. doi: 10.1038/s41467-022-35427-7 (PMC9813373; doi:10.1038/s41467-022-35427-7)
Supplement: Supplementary file 2 — Peer Review File [file 41467_2022_35427_MOESM2_ESM.pdf]

nature portfolio

Peer Review File

Spontaneous vortex formation by microswimmers with retarded attractions

Open Access This file is licensed under a Creative Commons Attribution 4.0 International License, which permits use, sharing, adaptation, distribution and reproduction in any medium or format, as long as you give appropriate credit to the original author(s) and the source, provide a link to the Creative Commons license, and indicate if changes were made. In the cases where the authors are anonymous, such as is the case for the reports of anonymous peer reviewers, author attribution should be to 'Anonymous Referee' followed by a clear attribution to the source work. The images or other third party material in this file are included in the article's Creative Commons license, unless indicated otherwise in a credit line to the material. If material is not included in the article's Creative Commons license and your intended use is not permitted by statutory regulation or exceeds the permitted use, you will need to obtain permission directly from the copyright holder. To view a copy of this license, visit http://creativecommons.org/licenses/by/4.0/.

REVIEWER COMMENTS

Reviewer #1 (Remarks to the Author):

The manuscript by Wang et al considers how rotation can spontaneously emerge due to delays in the sharing of information within an active system. The results include an experimental colloidal system activated using laser light, in which the direction of self-propulsion is programmed to change with a time delay. This leads to curious experimental observations in systems with a single active particle and a system with an active cluster, in which rotational states spontaneously emerge. The manuscipt also includes a detailed theory of how a single active particle behaves and a phenomenological theory describing the many-particle system.

The manuscript's novelty lies in creating an experimental model system made out of colloids to explore a common phenomenon of biological active systems (that is, the time delay required to process information). I find the strength of the manuscipt to be the detailed analysis of the single-particle case, including experiment, theory, and reduction to a minimal model. However, the many-particle case is explored at a more phenomenological level, which was in parts unclear to me. I also found that the implications of this research for the broader set of active-matter systems could be more clearly explained. I recommend that the authors address these major points, described in detail below.

For the many-particle phenomenology, could the authors relate their conclusions closer to the one-particle model? Is there a more quantitative many-particle model that could be developed based on the understanding of the one-particle system and the experimental observations?

For the broader implications of this work, the authors motivate their study based on a broad range of biological and synthetic systems. Could the authors elaborate more about the implication of their results for these systems? For example, for what phenomena can time-delay be neglected, and when is it crucial? What biological systems can the present colloidal set up be used to model most closely?

The authors contrast the mechanism for rotation in their system to non-reciprocal coupling in Ref.[51], but this comparison was unclear to me. Could the authors elaborate on the differences between these mechanisms and the emergence of rotation at the single-particle vs many-particle level?

For the single-particle system, in the main text the result of Eq.(4) is presented without much motivation. Although there is a detailed calculation in SI, I would suggest including some of the approach and intuition for Eq.(4) within the main text.

Reviewer #2 (Remarks to the Author):

This paper reports a combined experimental and theoretical study of a system of Brownian microswimmers exhibiting self-propulsion towards a target. Using feedback control, the set-up is such that the self-propulsion involves a well-defined time-delay, thereby mimicking retardation effects in systems due to retarded perception of information. The physics of active Brownian particle following certain "lab-designed" rules of motion is a currently a very lively field attracting researchers not only from soft and condensed matter physics, but also from biophysics and engineering. However, the impact of time delay has received less attention so far, although it is clearly an important ingredient in many real systems exchanging information. With its focus on the impact of delay, the paper here thus brings forward an important new aspect.

The article describes results for both, the single-particle behavior of the retarded microswimmer, and the cooperative behavior of several microswimmers. Indeed, already the single-particle motion is highly non-trivial in that there is a critical delay time, beyond which the particle circulates around the target. The authors analyse this effect very convincingly. Indeed, starting from the stochastic delay differential equation for the relevant angle and performing several approximations, they show that the observed instability of the single-particle motion can be explained as a bifurcation in an underlying double-well potential generated through the time delay. Moreover, when combining several particles, these rotational motions synchronise, yielding the "spontaneous vortex formation" motivating the title of the manuscript.

All in all, this is a very convincing and stimulating study. The described physics is novel and it adds an exciting contributing to the emerging field of designing and understanding "intelligent" particles as models for interactions and phenomena in real life. Moreover, the described research is carried out very carefully. In particular, the theoretical analysis is highly non-trivial, and the mapping to a particle in a double-well potential provides a very clever way to understand the occurring bifurcation. I therefore think that the manuscript is suitable for publication in Nature Communications. I have only a small number of points which the authors may consider:

- discussion of Figure 2A (bottom): the authors say that "the propulsion angle fluctuates around a stable non-zero value". To me it rather seems that the angle switches between two non-zero values ... !?

- it is not entirely clear how one comes from Eq (3) in the main text to the stochastic equation in the last paragraph page 6.

- Can the authors comment on the question, whether and to which extent the overall synchronisation of motion in the ensemble depends on the number of particles considered?

- Given that the BD simulations of the many-body system yield somewhat different results, the authors conclude that hydrodynamical interactions (neglected in BD) play a significant role. However, I would expect that also the shape of repulsion could play a role. Can the authors comment on this point?

- I did not quite understand a statement in the discussion (second paragraph from bottom on page 13), where the authors sat that their system provides a "microscopic underpinning" for vision-cone models. What exactly is meant here? Is it the underlying "nonreciprocity" of the couplings .... ?

```
--------------------------------------------------------------------------------
Response to the Report of the First Referee
--------------------------------------------------------------------------------
```

The manuscript by Wang et al considers how rotation can spontaneously emerge due to delays in the sharing of information within an active system. The results include an experimental colloidal system activated using laser light, in which the direction of self-propulsion is programmed to change with a time delay. This leads to curious experimental observations in systems with a single active particle and a system with an active cluster, in which rotational states spontaneously emerge. The manuscript also includes a detailed theory of how a single active particle behaves and a phenomenological theory describing the many-particle system.

The manuscript's novelty lies in creating an experimental model system made out of colloids to explore a common phenomenon of biological active systems (that is, the time delay required to process information). I find the strength of the manuscript to be the detailed analysis of the single-particle case, including experiment, theory, and reduction to a minimal model. However, the many-particle case is explored at a more phenomenological level, which was in parts unclear to me. I also found that the implications of this research for the broader set of active-matter systems could be more clearly explained. I recommend that the authors address these major points, described in detail below.

We thank the reviewer for the careful evaluation of our work and the helpful questions and suggestions. We hope that the revised version clarifies the raised issues to the reviewer's satisfaction.

For the many-particle phenomenology, could the authors relate their conclusions closer to the one-particle model? Is there a more quantitative many-particle model that could be developed based on the understanding of the one-particle system and the experimental observations?

In the revised version, we have rewritten the corresponding paragraphs, which are now referring to a new figure S9 in the SI, comparing data obtained for various numbers of particles. It shows that the bifurcation diagram of the single-particle model remains valid for to up to 6 particles (one complete shell). This holds exactly in the simulation and, within measurement errors, also in the experiment. For larger numbers of particles (several shells), it still holds approximately if one accounts for the increasing orbit radius, as shown in Fig. 4B (experiment for 15 particles) and for the simulations in Fig. S9D (14-69 particles). The mismatch between the experiments and simulations observed for larger particle numbers seems to arise from hydrodynamic backflow effects from the propulsion mechanism. We crudely accounted for them in some of our simulations as well as in an ad-hoc extension of the single-particle theory, together with an empirical backflow-induced bias, as detailed in the SI. Thereby, we can roughly account for the experimentally observed co- and counter-rotating states, as shown in the newly added figure S10. Unfortunately, the (crudely) extended simulations constitute

the only "more quantitative" useful model we can currently provide. We think that this level of detail is appropriate for the present purpose and leave the full-fledged numerical replication of all experimental details to future work.

For the broader implications of this work, the authors motivate their study based on a broad range of biological and synthetic systems. Could the authors elaborate more about the implication of their results for these systems? For example, for what phenomena can time-delay be neglected, and when is it crucial? What biological systems can the present colloidal set up be used to model most closely?

We added a table with measured delay times for animals to the SI, where we point out that for delay times comparable to characteristic time scales of an external stimulus, qualitative behavior similar to that paradigmatically studied in the present work may be expected. Which of these examples comes closest to our analysis may of course depend on many quantitative details that we cannot anticipate, but bacteria should be the nearest and most direct natural examples, because they are of similar size to our particles and also operate in an aqueous thermal environment. However, see also the videos (Video1, Video2), for the same robust phenomenology, on a macroscopic scale.

The authors contrast the mechanism for rotation in their system to non-reciprocal coupling in Ref.[51], but this comparison was unclear to me. Could the authors elaborate on the differences between these mechanisms and the emergence of rotation at the single-particle vs many-particle level?

We apologize for the confusion caused and have revised the formulation to make it more comprehensible. As now stated in the main text, the non-reciprocal coupling reported in Ref. [51] requires many-body effects for stabilizing the chiral phases, since noise would destroy the chiral phase for two non-reciprocally coupled particles. This is in contrast to our observation that a particle coupled by a delayed attraction to a target could reveal a robust rotation without many-body effects. We argue that a possible interpretation might come from the infinite number of relaxation modes encoded in a time-delayed system.

For the single-particle system, in the main text the result of Eq.(4) is presented without much motivation. Although there is a detailed calculation in SI, I would suggest including some of the approach and intuition for Eq.(4) within the main text.

Thank you very much for the suggestion! We have expanded the explanation of the derivation in the main text.

`Response to the Report of the Second Referee`

This paper reports a combined experimental and theoretical study of a system of Brownian microswimmers exhibiting self-propulsion towards a target. Using feedback control, the set-up is such that the self-propulsion involves a well-defined time-dwelay, thereby mimicking retardation effects in systems due to retarded perception of information. The physics of active Brownian particle following certain "lab-designed" rules of motion is a currently a very lively field attracting researchers not only from soft and condensed matter physics, but also from biophysics and engineering. However, the impact of time delay has received less attention so far, although it is clearly an important ingredient in many real systems exchanging information. With its focus on the impact of delay, the paper here thus brings forward an important new aspect.

The article describes results for both, the single-particle behavior of the retarded microswimmer, and the cooperative behavior of several microswimmers. Indeed, already the single-particle motion is highly non-trivial in that there is a critical delay time, beyond which the particle circulates around the target. The authors analyse this effect very convincingly. Indeed, starting from the stochastic delay differential equation for the relevant angle and performing several approximations, they show that the observed instability of the single-particle motion can be explained as a bifurcation in an underlying double-well potential generated through the time delay. Moreover, when combining several particles, these rotational motions synchronise, yielding the "spontaneous vortex formation" motivating the title of the manuscript.

All in all, this is a very convincing and stimulating study. The described physics is novel and it adds an exciting contributing to the emerging field of designing and understanding "intelligent" particles as models for interactions and phenomena in real life. Moreover, the described research is carried out very carefully. In particular, the theoretical analysis is highly non-trivial, and the mapping to a particle in a double-well potential provides a very clever way to understand the occurring bifurcation. I therefore think that the manuscript is suitable for publication in Nature Communications. I have only a small number of points which the authors may consider:

We thank the reviewer for the careful evaluation of our work and the helpful questions and suggestions. We hope that the revised version clarifies the raised issues to the reviewer's satisfaction and is now recommendable for publication.

- discussion of Figure 2A (bottom): the authors say that "the propulsion angle fluctuates around a stable non-zero value". To me it rather seems that the angle switches between two non-zero values ... !?

We have corrected the wording to “transiently fluctuates around”.

- it is not entirely clear how one comes from Eq (3) in the main text to the stochastic equation in the last paragraph page 6.

Thank you very much for the comment! We have expanded the explanation of the derivation in the main text.

- Can the authors comment on the question, whether and to which extent the overall synchronisation of motion in the ensemble depends on the number of particles considered?

We included a new figure Fig. S9 into the SI which shows that including new particles exponentially decreases the transition probability between the two rotating states. The data were obtained from a simulation which did not take into account hydrodynamic effects. Unfortunately, the exponentially increasing waiting time makes obtaining the corresponding experimental results almost impossible.

- Given that the BD simulations of the many-body system yield somewhat different results, the authors conclude that hydrodynamical interactions (neglected in BD) play a significant role. However, I would expect that also the shape of repulsion could play a role. Can the authors comment on this point?

In newly added figure S10, we present phase diagrams for the appearance of co- and counter-rotating shells obtained from our simulations extended by ad-hoc “back-flow-induced interactions” decaying with the inverse distance between the particles and from experiments. The demonstrated agreement between the simulated and experimental results suggests that the observed phenomena are robust against the details of the shape of the back-flow. This conclusion is further supported by the fact that the prediction of our phenomenological theory fits the phase boundary in the two diagrams almost perfectly. Finally, to highlight that the interparticle interactions are not necesserily only of hydrodynamic origin, we also now mention the mutually induced phoretic contributions to the interactions in the abstract.

- I did not quite understand a statement in the discussion (second paragraph from bottom on page 13), where the authors sat that their system provides a “microscopic underpinning” for vision-cone models. What exactly is meant here? Is it the underlying “nonreciprocity” of the couplings .... ?

We have now slightly shifted and rewritten this sentence, which unfortunately was indeed not very clear and was not intended to imply a major role of non-reciprocity, per se (although the mentioned interaction rules are indeed non-reciprocal). We hope that the revision is now

clearer and more precise.

REVIEWERS' COMMENTS

Reviewer #1 (Remarks to the Author):

The authors have addressed my comments well. The authors have clarified the manuscript and I am happy to recommend publication.

Reviewer #2 (Remarks to the Author):

In the revised version of the manuscript and the accompanying letter, the authors have convincingly addressed all aspects raised in my earlier report. I particularly acknowledge the inclusion of new results on the dependency on N and the shape of the particles, which clearly show the robustness of the main messages of the manuscript.

I am therefore happy to recommend publication of the revised manuscript.
